# Supplementary material for: Task Demands Predict a Dynamic Switch in the Content of Awake Hippocampal Replay
Source: Neuron. 2017 Nov 15;96(4):925–935.e6. doi: 10.1016/j.neuron.2017.09.035 (PMC5697915; doi:10.1016/j.neuron.2017.09.035)
Supplement: Document S1. Figures S1–S14 and Table S1 [file mmc1.pdf]

Neuron, Volume *96*

Supplemental Information

Task Demands Predict a Dynamic Switch in the Content of Awake Hippocampal Replay

H. Freyja Ólafsdóttir, Francis Carpenter, and Caswell Barry

Animal	Experimental Day	#Place cells	Outbound error (cm)	Inbound error (cm)	Mean Error (cm)	% Replay	p-value (KS test, data vs shuffle)
R2142	4	37	22	10	17	37.54	1.05×10^{-29}
	5	46	20	12	16	24.16	6.52×10^{-19}
	6	39	30	24	27	46.34	7.29×10^{-5}
R2192	4	34	20	16	18	40.46	1.21×10^{-34}
	5	39	16	12	14	39.64	8.12×10^{-83}
	6	70	12	12	12	43.09	1.77×10^{-55}
	7	68	10	10	10	56.25	6.77×10^{-45}
	8	72	10	10	10	59.35	2.32×10^{-32}
R2198	9	30	20	12	16	32.26	1.72×10^{-50}
	10	27	12	12	12	29.79	4.61×10^{-8}
R2217	5	25	16	16	16	23.13	1.81×10^{-12}
	7	40	14	18	16	31.00	5.25×10^{-12}
R2242	1	30	18	22	18	27.43	4.03×10^{-36}
	4	21	24	18	22	70.00	0.00072
R2335	3	34	30	26	28	11.11	4.44×10^{-6}
R2336	2	25	24	20	22	19.86	3.45×10^{-9}
	3	39	24	26	26	45.90	3.76×10^{-10}
	5	34	16	28	20	7.70	0.0013
	6	67	12	14	14	39.67	2.28×10^{-13}
R2337	2	50	22	14	18	27.82	3.97×10^{-37}
	3	50	20	16	18	40.35	5.88×10^{-32}
	4	71	18	16	16	40.84	1.98×10^{-75}
	5	48	28	22	24	44.81	3.58×10^{-59}
	6	46	26	20	22	23.53	3.58×10^{-20}
Median		34	20	16	17.5	38.59	

Table S1. Related to Figure 1. Place Cell Number, Decoding Errror and Replay for Each Experimental Day

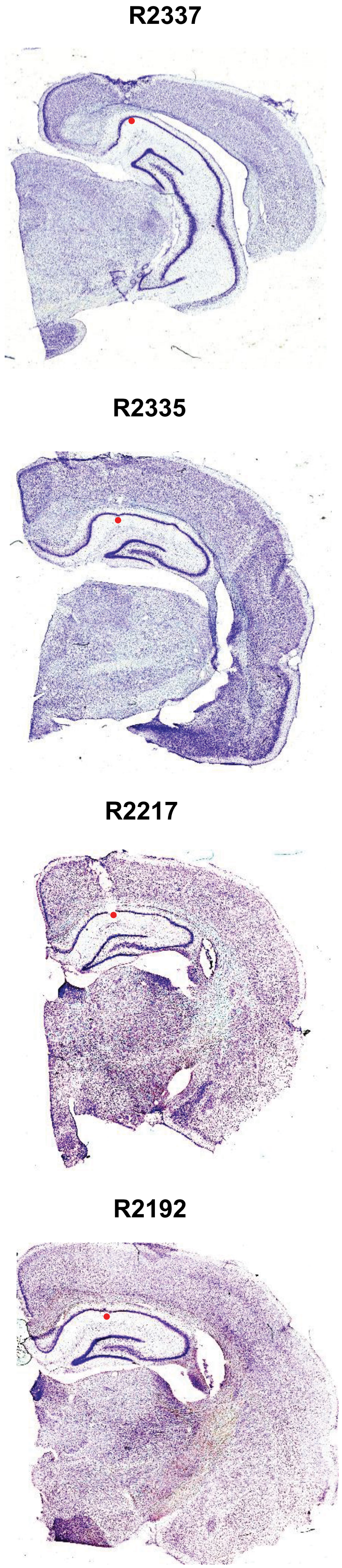

Figure S1. Related to Figure 1. Tetrode Locations. Four representative examples of Cresyl violet stained sections showing the tetrode tracks from coronal sections. Red circle indicates the recording location for data included in this study. Title shows rat ID.

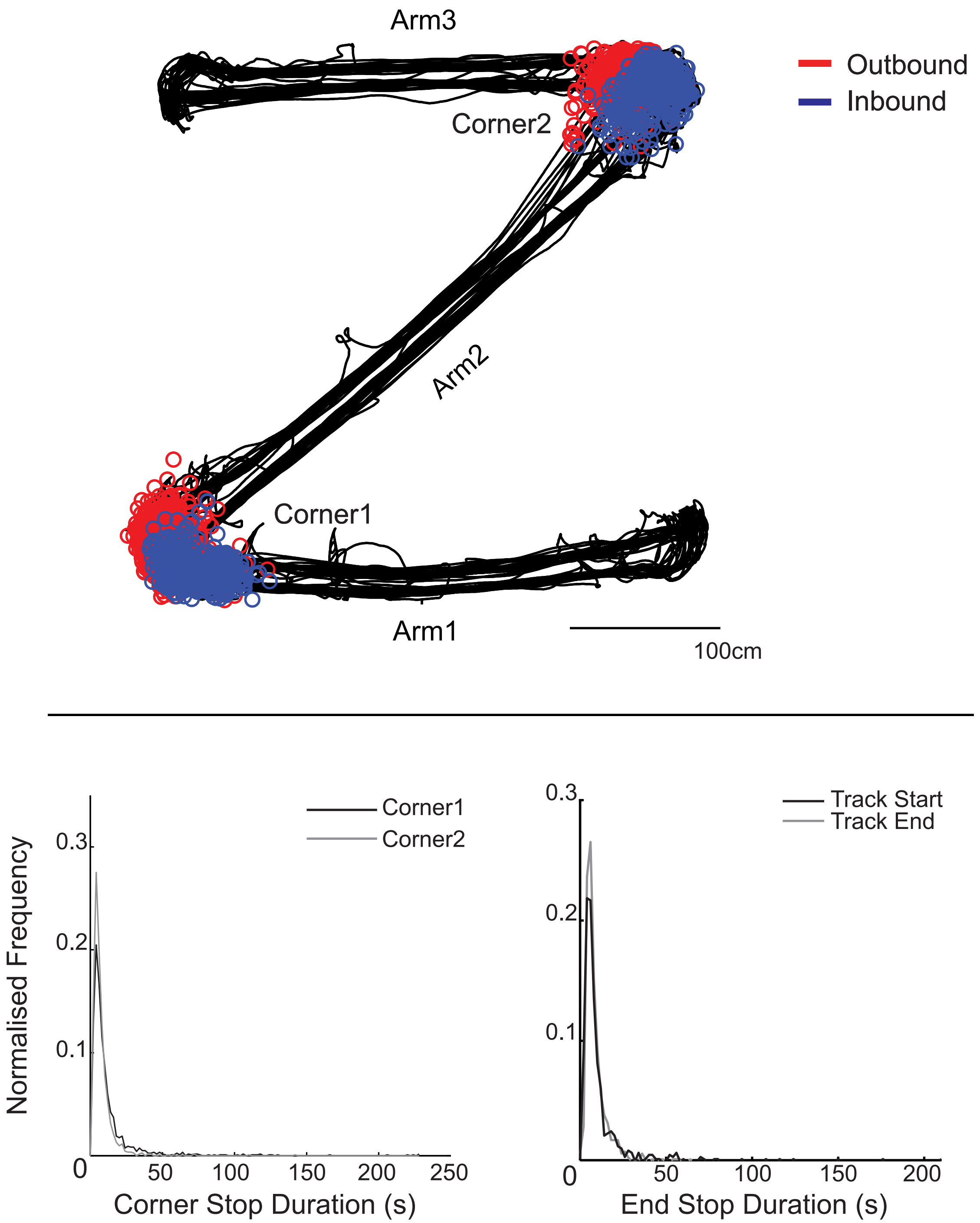

Figure S2. Related to Figure 1. Position and Duration of Corner Stops. Top: The location of animals during corner periods for outbound (Arm1 -> Arm3) and inbound (Arm3 -> Arm1) runs are indicated by the coloured circles. Each circle indicates mean position during a single corner period. Black line - path from lone recording session (i.e. from a single animal), included to illustrate the track extent. Note, primary tracking LED was offset slightly (i.e. 7cm) from the side of animals' heads - hence, outbound and inbound runs appear to not overlap. Bottom: Left: distribution of stopping period durations at the two corners. Right: distribution of stopping period durations at the two track ends (track start = beginning of arm1; track end = end of arm3).

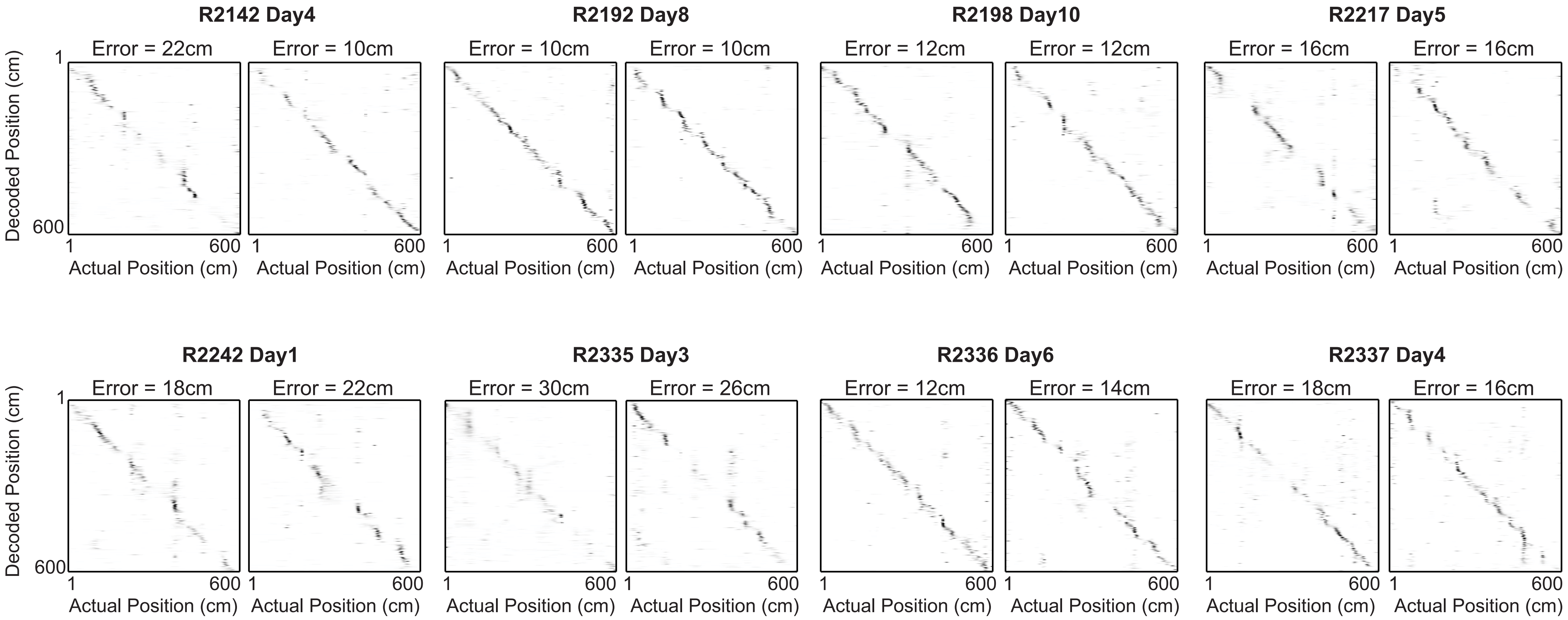

Figure S3. Related to Figure 1. Accuracy of Online Decoding. Confusion matrices for position decoding carried out on spike data while the animals ran (>10cm/s) on the Z-track. A representative session was selected for each animal. Right: confusion matrices for outbound runs, left: confusion matrices for inbound runs. Darker shades indicate higher probability of position. x-axis: actual position of rat (cm), y-axis: decoded position (cm).

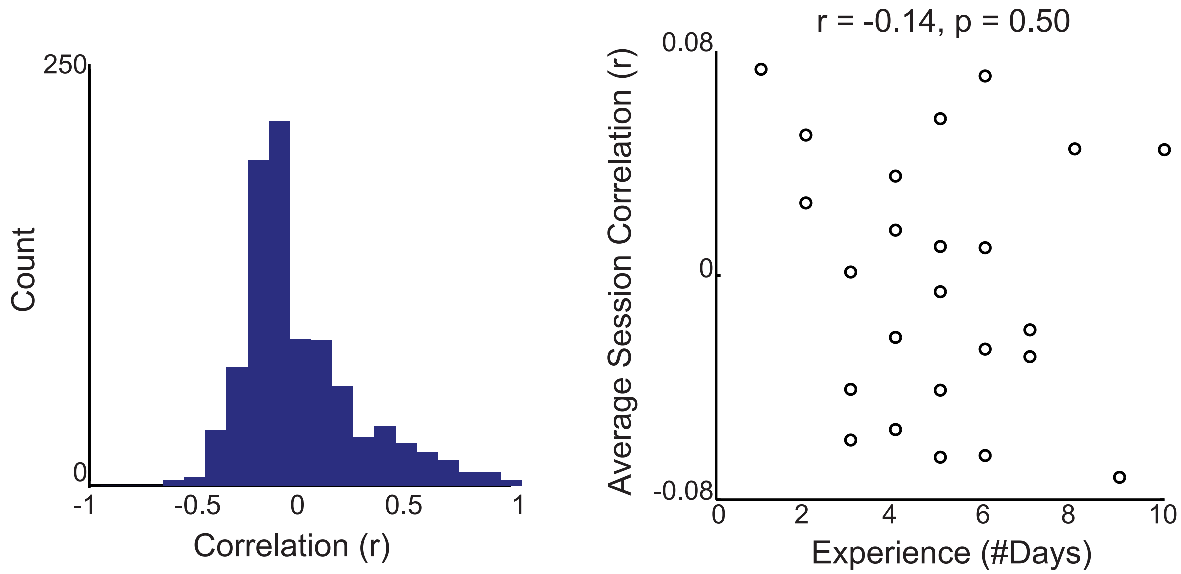

Figure S4. Related to Figure1. Place Cell Activity Is Directionally Modulated on the Z-track. Left: distribution of spatial correlations (r) between inbound and outbound ratemaps for all cells recorded on the track. Right: Mean spatial correlation for each session vs. animals' experience of the track.

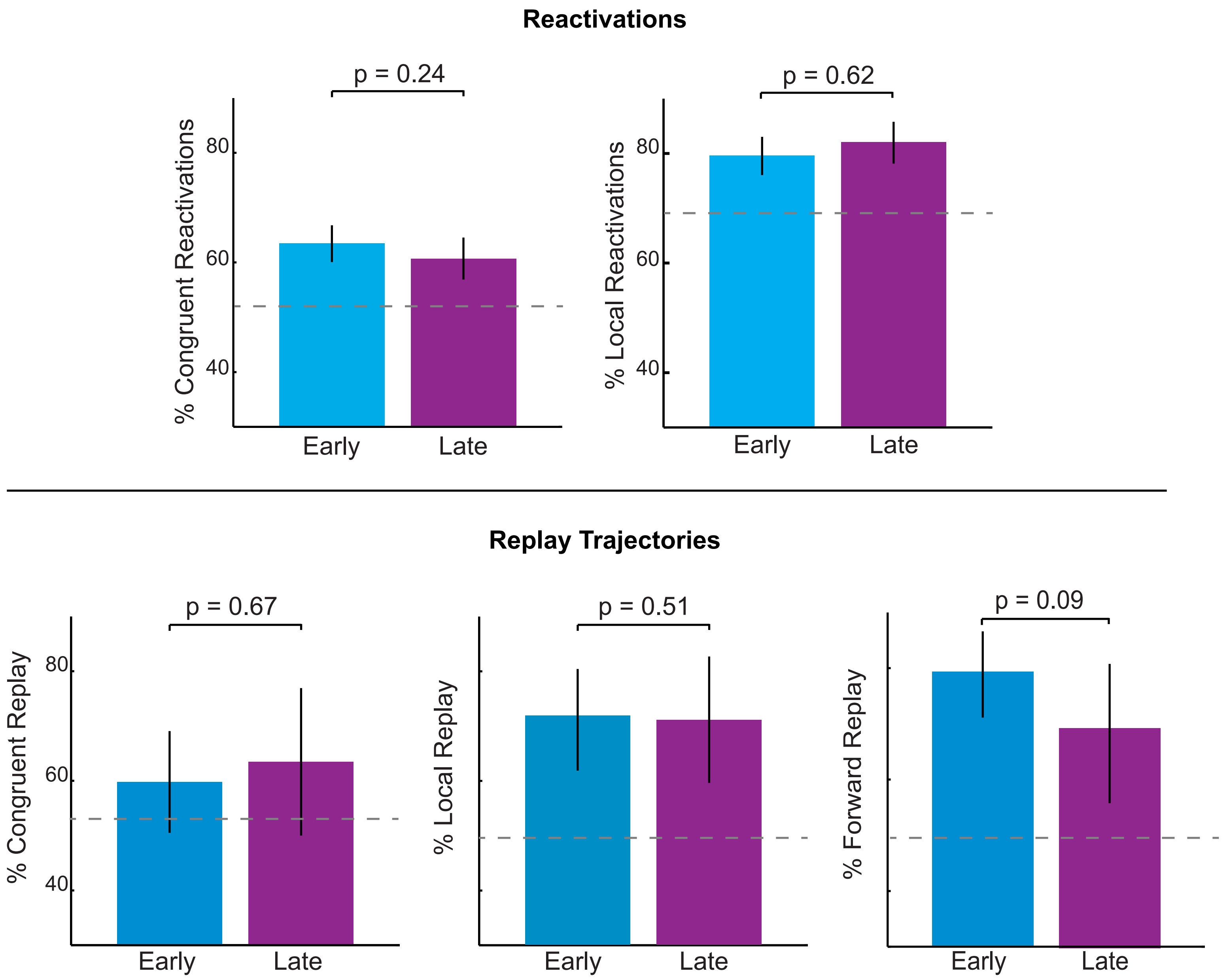

Figure S5. Related to Figure 2. Replay for Early and Late Engaged Periods of Corner Stops. Upper panel: Proportion of reactivation events classified as congruent (left) or local (right) for early (blue) and late (purple) temporal sections of engaged periods at corner stops. Lower panel: proportion of replay events classified as congruent (left), local (middle) or forward (right) for early and late temporal sections. Error bars indicate 95% confidence interval based on bootstrapped data. Dashed horizontal line shows chance.

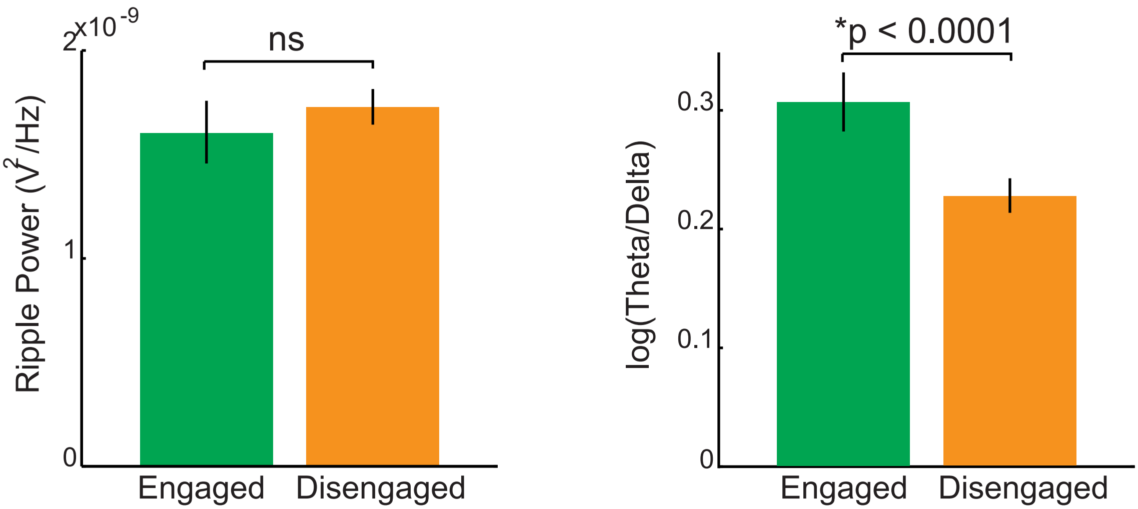

Figure S6. Related to Figure 2. Power in the Theta and Ripple Band During Reactivations. Left: mean power in the ripple band (150-250Hz) for engaged (green) and disengaged (amber) events. Right: same as left but for log(theta/delta) ratio, where theta band is 6-12Hz and delta band is 2-4Hz. Error bars show 95% confidence interval based on bootstrapped data.

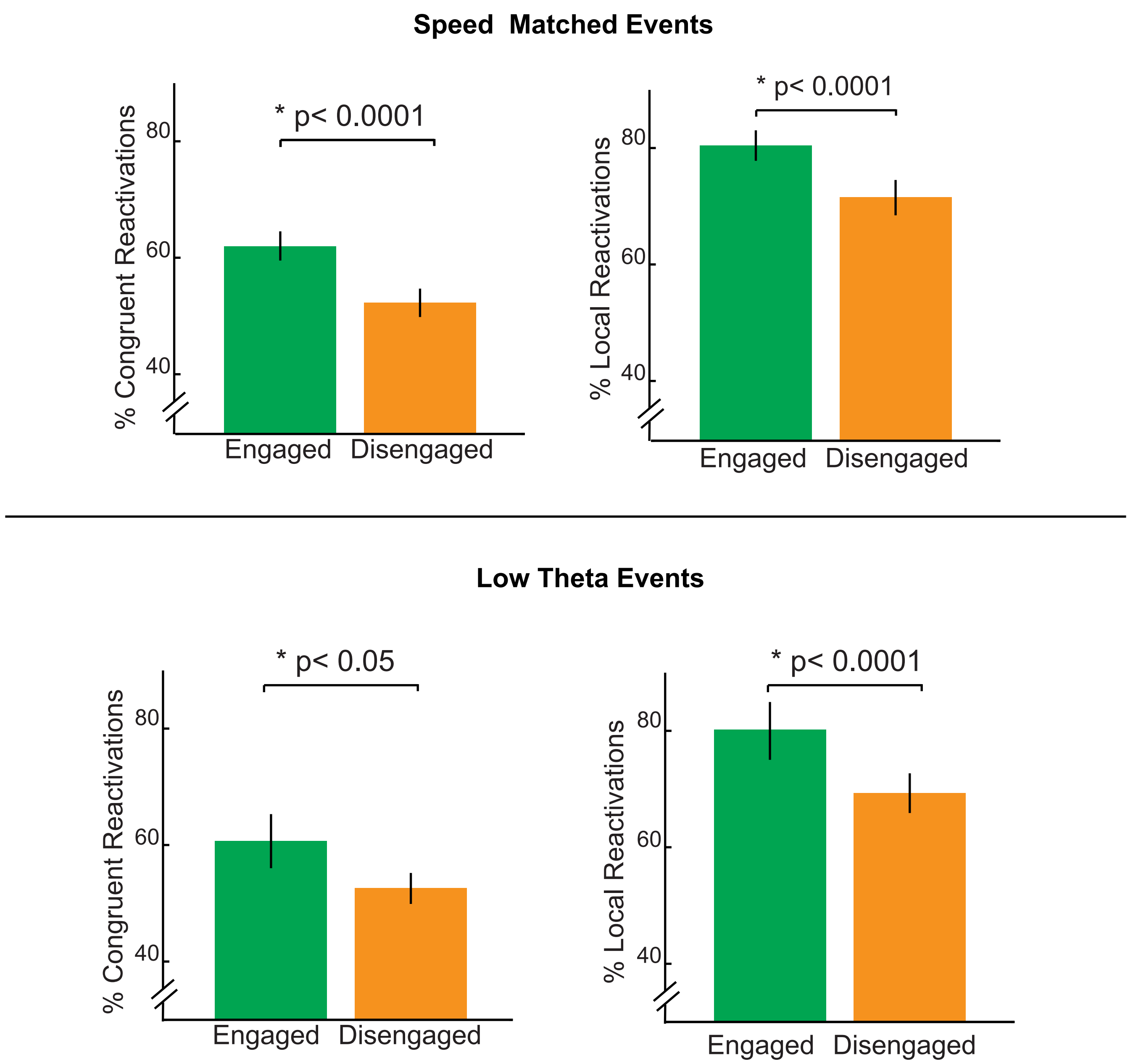

Figure S7. Related to Figure 2. Effect of Task Engagement Over Reactivations is Not an Artefact of Speed or High Theta During Events. Upper panel(left): Proportion of events classified as congruent for engaged (green) and disengaged (amber) periods after excluding events in order to match the median speeds of the two categories. Right: same as left but for local reactivations. Before excluding events median engaged speed = 1.80cm/s, median disengaged speed = 1.13cm/s. Bottom panel: same as upper but after the exclusion of events with high theta power (i.e. theta power < mean theta power during movement -1SD, mean = 0.64, SD = 0.42, threshold = 0.22). Mean engaged log(theta/delta) power = 0.31, mean disengaged log(theta/delta) power = 0.23. Error bars show 95% confidence interval based on bootstrapped data.

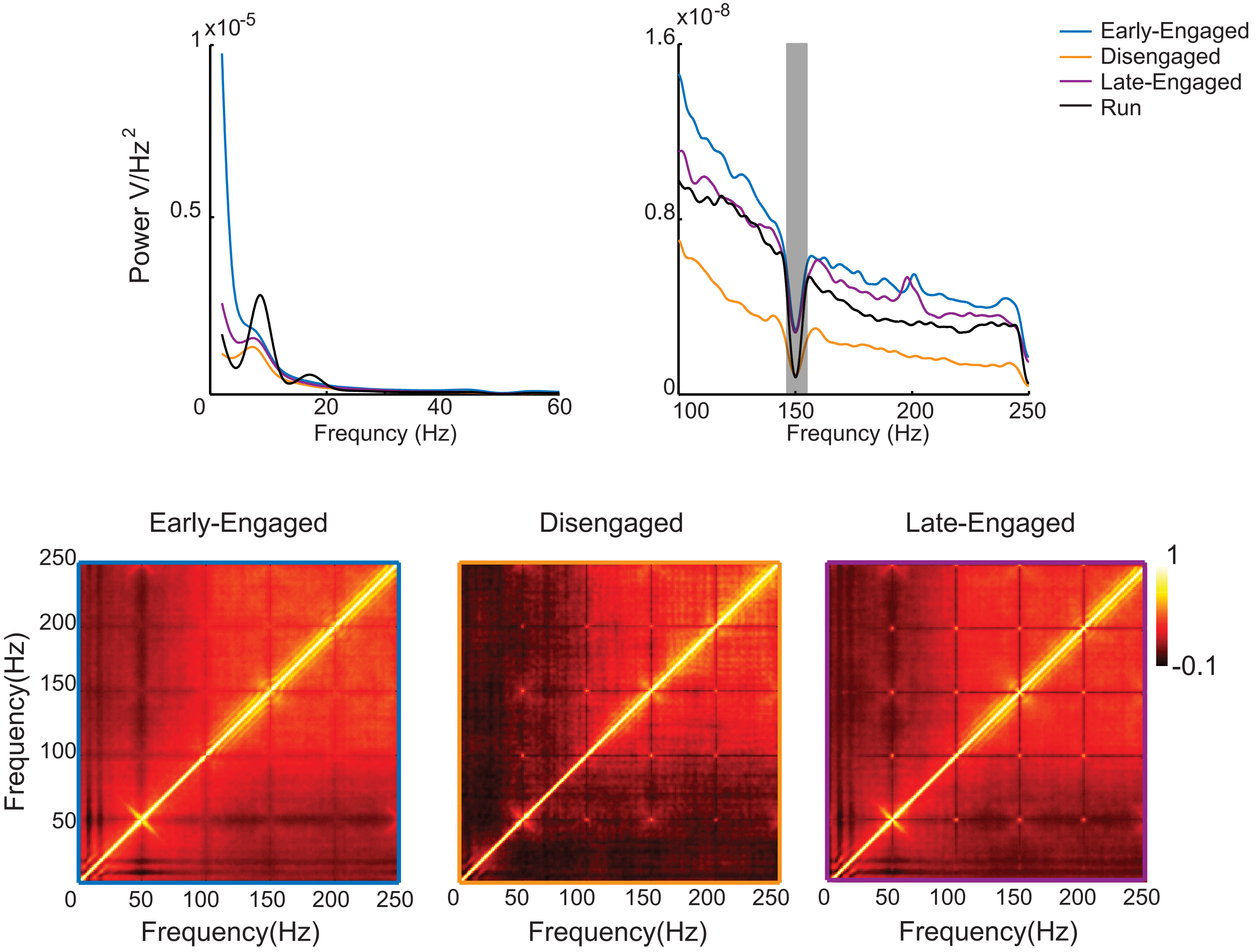

Figure S8. Related to Figure 2. Power-Spectral Density (PSD) and Self-Coherence Analysis for Distinct Temporal Sections of Corner Stops. Top left: PSD for early-engaged (blue), disengaged (amber), and late-engaged (purple) sections of corner stops as well as during periods of movement (black) for low (1-60Hz,left) and high (100-250Hz, right) frequencies. Bottom: self-coherence (Masimore et al. 2004) plots for the three temporal sections at the corners. Lighter colours indicate higher correlations. A notch filter was applied at multiples of 50Hz to eliminate electrical mains noise.

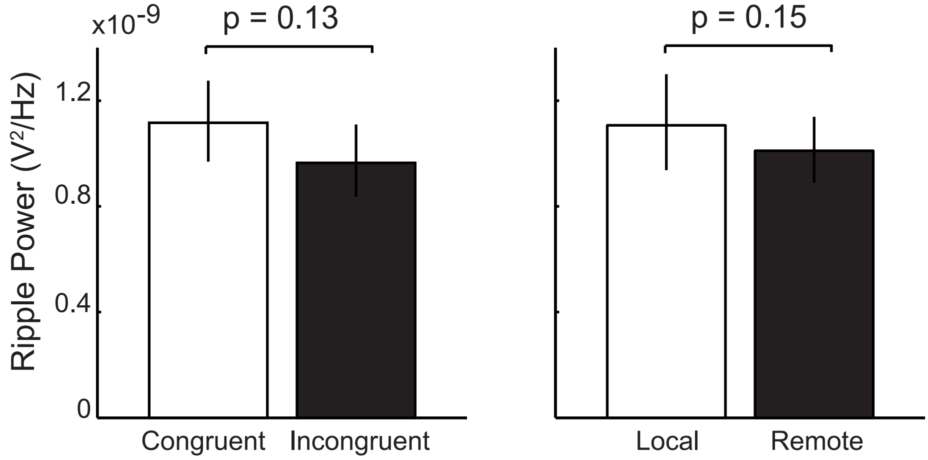

Figure S9. Related to Figure2. Ripple Power Does Not Differ Between Reactivation Categories. Power in the ripple band (150-250Hz) for congruent and incongruent reactivation events (left) and local and remote (right) events. Error bars indicate 95% confidence interval based on bootstrapped data.

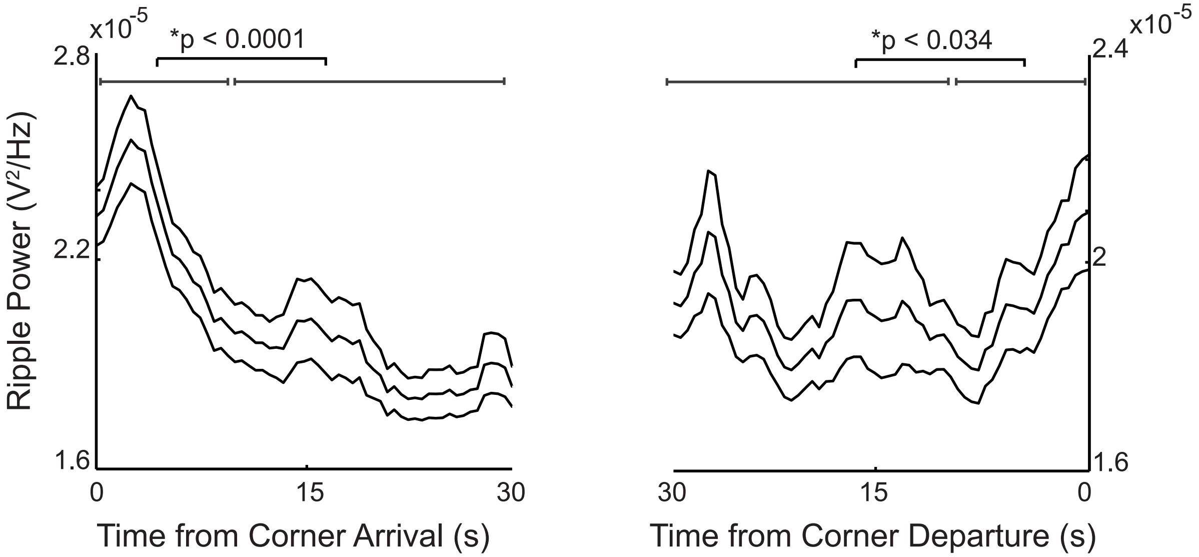

Figure S10. Related to Figure 2. Ripple Power During Corner Stops. Power in the LFP ripple band (150-250Hz) as a function of time from arrival (left) and departure (right) from a corner. x-axis: time (s), y-axis: ripple power (V^2/Hz). Power in the first 10 seconds after arrival and before departure was higher than power in the 20-30 second period. Error bars show 1SD of the data.

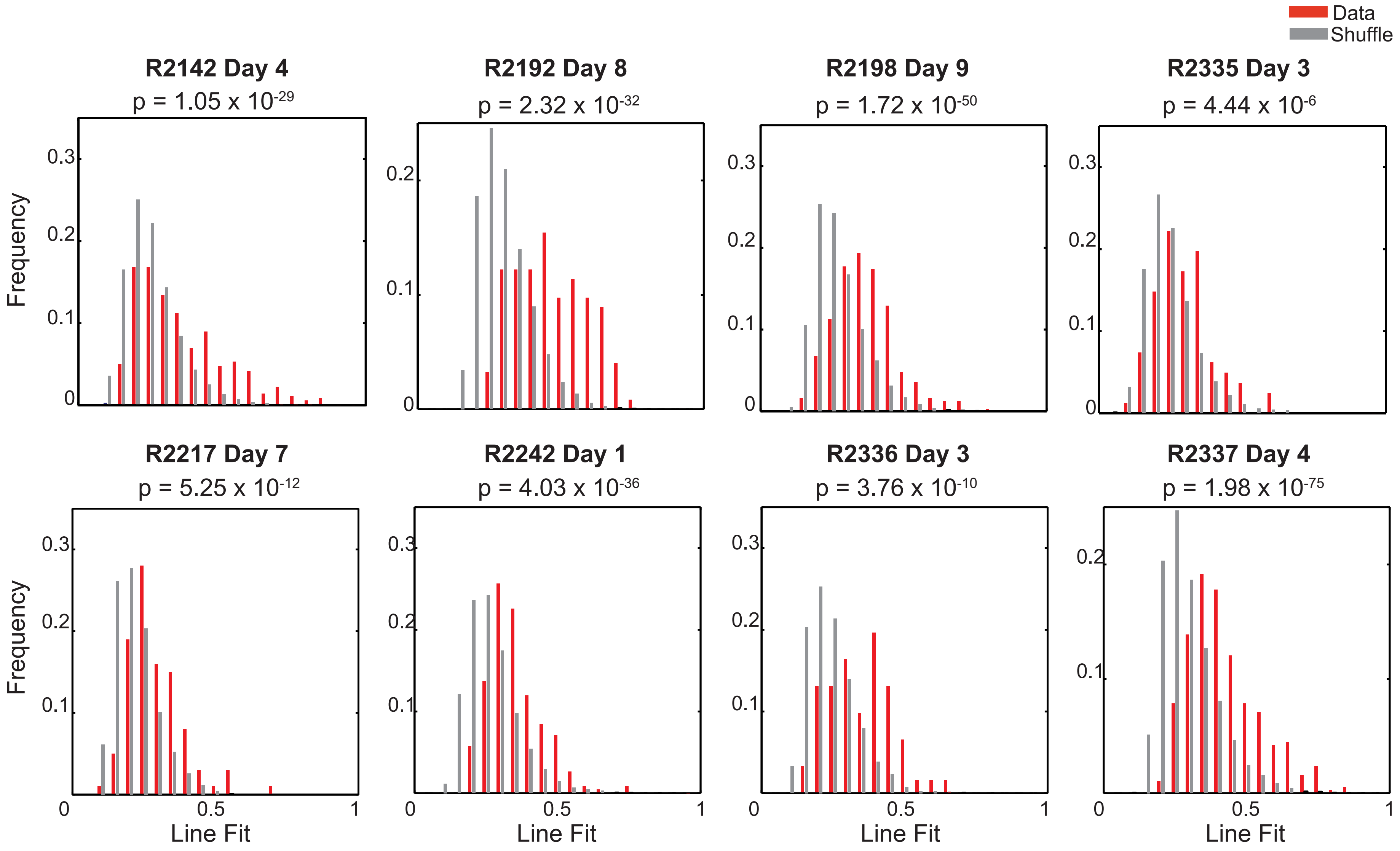

Figure S11. Related to Figure 3. Histogram of Best-Fit Lines for Replay Events. Representative examples, from each animal, of frequency distributions of best-fit lines for replay events recorded in a given session (red) against that for shuffled data (grey). Title shows p-value of a Kolmogorov-Smirnov test comparing the data and shuffle distributions. The data and shuffle distributions were normalised by dividing by the total number of events in each.

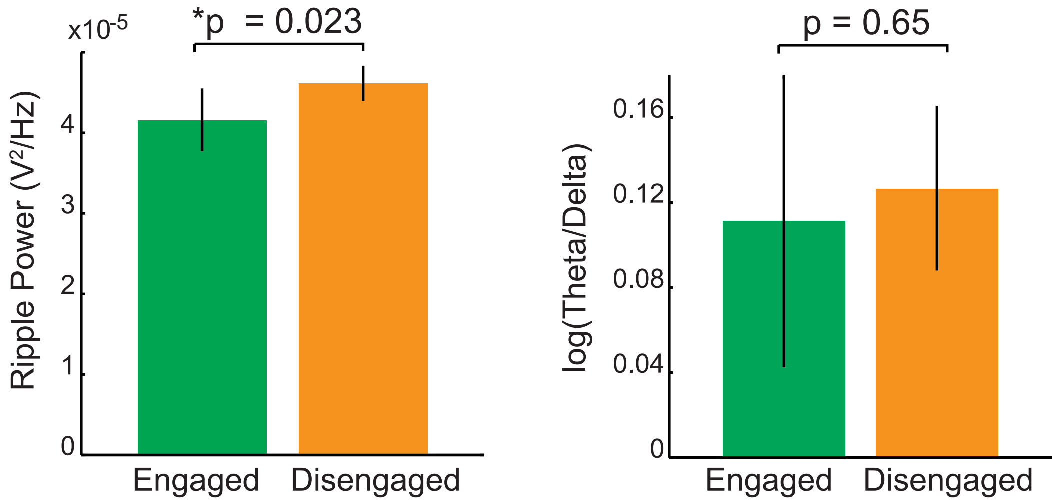

Figure S12. Related to Figure 3. Ripple and Theta Power During Replay Events. Left: Mean power in the ripple band (150-250Hz) during engaged (green) and disengaged (amber) replay events. Right: same as left but for log(theta/delta) ratio, where theta band is 6-12Hz, and delta band 2-4Hz. Error bars indicate 95% confidence interval based on bootstrapped data.

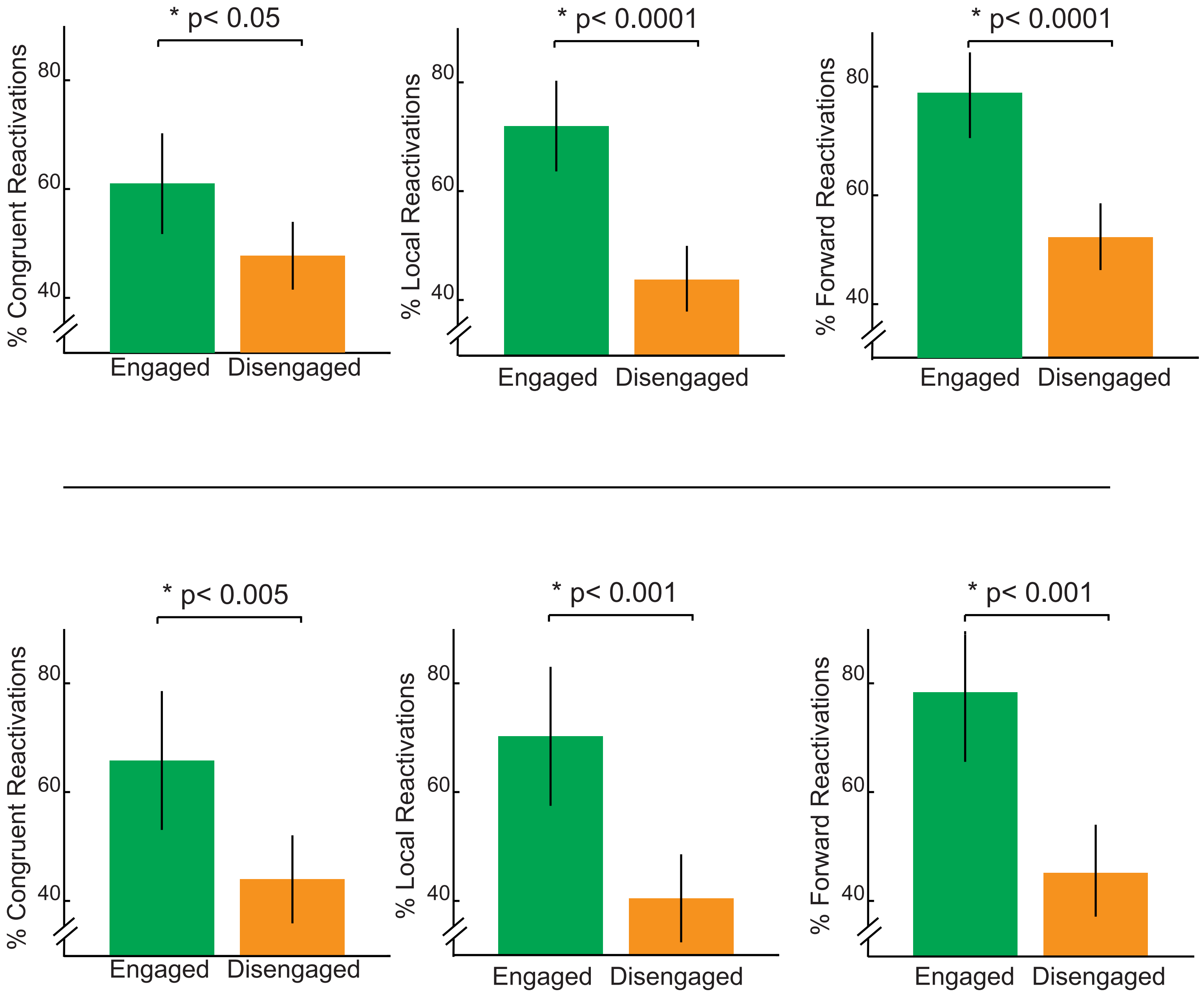

Figure S13. Related to Figure 3. Effect of Task Engagement on Replay After Limiting Analysis to Long Trajectory Events and Events Co-Occurring with Ripples. Upper panel**:** Proportion of events classified as congruent (left), local (middle) or forward (right) for engaged (green) and disengaged (amber) periods after excluding events with a shallow (replay trajectories >2m long) slope (mean engaged slope = 8.7m/s, mean disengaged slope = 7.7m/s). Lower panel: same as upper panel but once analysis was limited to events co-occurring with ripples (38.84% of engaged events, 40.12% of disengaged events). Error bars indicate 95% confidence interval based on bootstrapped data.

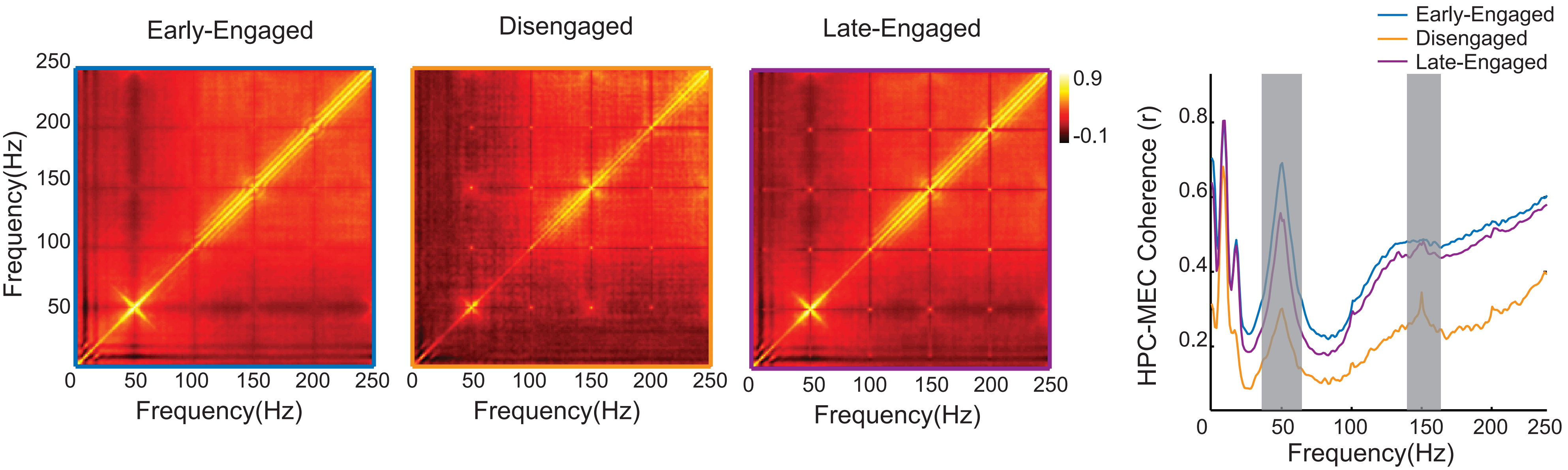

Figure S14. Related to Figure 4. Hippocampal-MEC LFP Coherence During Engaged and Disengaged Replay. Left: Cross-coherence (Masimore et al. 2004) between hippocampal and MEC LFP during early-engaged, disengaged and late-engaged sections of corner stops. Lighter colours indicate higher correlations. Right: Hippocampal-MEC LFP coherence during the engaged-early (blue), disengaged (amber) and engaged-late (purple) sections. Grey area covers a portion of the LFP band contaminated by mains (50Hz) noise.
