# Supplementary material for: Task Demands Predict a Dynamic Switch in the Content of Awake Hippocampal Replay
Source: Neuron. 2017 Nov 15;96(4):925–935.e6. doi: 10.1016/j.neuron.2017.09.035 (PMC5697915; doi:10.1016/j.neuron.2017.09.035)
Supplement: Document S2. Article plus Supplemental Information [file mmc2.pdf]

Article

Neuron

Task Demands Predict a Dynamic Switch in the Content of Awake Hippocampal Replay

Highlights

- Place cell replay content varies between periods of task engagement and disengagement
- Upon arrival/before departure from reward sites, replay depicts task-relevant places
- No such bias was observed during periods of extended immobility
- Grid cells from deep MEC were coherent with place cells during extended stops

Authors

H. Freyja Ólafsdóttir,
Francis Carpenter, Caswell Barry

Correspondence

h.olafsdottir@ucl.ac.uk (H.F.Ó.),
caswell.barry@ucl.ac.uk (C.B.)

In Brief

Ólafsdóttir et al. found that, upon arrival at and before departure from reward sites, but not during immobility, hippocampal replay depicts places related to current task demands. Occurrence of task-focused replay predicts decision accuracy. Grid cells were only coherent with replay during immobility.

Ólafsdóttir et al., 2017, Neuron *96*, 925–935
November 15, 2017 © 2017 The Authors. Published by Elsevier Inc.
https://doi.org/10.1016/j.neuron.2017.09.035

CellPress

Task Demands Predict a Dynamic Switch in the Content of Awake Hippocampal Replay

H. Freyja Ólafsdóttir,[1,3,*] Francis Carpenter,[1,2] and Caswell Barry[1,*]
[1]Research Department of Cell and Developmental Biology, UCL, Gower Street, London WC1E 6BT, UK
[2]Institute of Neurology, UCL, Queen Square, London WC1N 3BQ, UK
[3]Lead Contact
*Correspondence: h.olafsdottir@ucl.ac.uk (H.F.Ó.), caswell.barry@ucl.ac.uk (C.B.)
https://doi.org/10.1016/j.neuron.2017.09.035

SUMMARY

Reactivation of hippocampal place cell sequences during behavioral immobility and rest has been linked with both memory consolidation and navigational planning. Yet it remains to be investigated whether these functions are temporally segregated, occurring during different behavioral states. During a self-paced spatial task, awake hippocampal replay occurring either immediately before movement toward a reward location or just after arrival at a reward location preferentially involved cells consistent with the current trajectory. In contrast, during periods of extended immobility, no such biases were evident. Notably, the occurrence of task-focused reactivations predicted the accuracy of subsequent spatial decisions. Additionally, during immobility, but not periods preceding or succeeding movement, grid cells in deep layers of the entorhinal cortex replayed coherently with the hippocampus. Thus, hippocampal reactivations dynamically and abruptly switch between operational modes in response to task demands, plausibly moving from a state favoring navigational planning to one geared toward memory consolidation.

INTRODUCTION

Prominent theories of hippocampal function place it at the center of networks supporting memory and navigation (O'Keefe and Nadel, 1978; Scoville and Milner, 1957). The principal cell of the hippocampus is the place cell, whose activity during locomotion encodes the animal's self-location via spatially localized firing fields (place fields) (O'Keefe and Dostrovsky, 1971). However, during non-rapid eye movement (non-REM) sleep and pauses in locomotion, when sharp-wave ripple complexes (SWRs) transiently dominate the hippocampal local field potential (LFP) (Buzsáki et al., 1992; O'Keefe and Nadel, 1978), place cell activity de-couples from the animal's current location, reactivating past or future spatial trajectories (replay) (Foster and Wilson, 2006; Lee and Wilson, 2002; Wilson and McNaughton, 1994).

At the time of discovery, replay was proposed as the mechanism supporting systems-level memory consolidation (Wilson and McNaughton, 1994), the process by which memories are transferred out of the hippocampus, becoming less susceptible to hippocampal damage (Marr, 1971; Scoville and Milner, 1957). Consistent with this hypothesis, replay typically reflects recent experiences, particularly novel ones (Cheng and Frank, 2008; Foster and Wilson, 2006; O'Neill et al., 2008; van de Ven et al., 2016), is dependent on the NMDA receptor (Dupret et al., 2010; Silva et al., 2015), and is associated with cortical reactivations (Ji and Wilson, 2007; Rothschild et al., 2017; Wierzynski et al., 2009). Indeed, cortical replay has been found to temporally lag the hippocampus (Ólafsdóttir et al., 2016; Rothschild et al., 2017), suggestive of information flow from the hippocampus to the cortex (Ólafsdóttir et al., 2016; Rothschild et al., 2017). Furthermore, numerous studies have shown that cortical LFP patterns associated with sleep, such as delta waves (Maingret et al., 2016; Mednick et al., 2013) and spindles (Johnson et al., 2010), are temporally coordinated with SWRs (Battaglia et al., 2004; Peyrache et al., 2011; Sirota et al., 2003), and they have indicated that cortico-hippocampal dialogue may be important for learning (Maingret et al., 2016). More generally, SWRs originate in the hippocampus (Buzsáki, 2015; Suzuki and Smith, 1985), propagate into the cortex (Chrobak and Buzsáki, 1994, 1996), and occur at a greater rate after learning (Eschenko et al., 2008). Elimination of SWRs during rest impairs spatial learning (Ego-Stengel and Wilson, 2010; Girardeau et al., 2009). Conversely, hippocampal reactivation during rest enhances learning (de Lavilléon et al., 2015; Rasch et al., 2007).

Nevertheless, it is now apparent that replay, and the roles attributed to it, are more diverse than first thought. While the role of replay during non-REM sleep (offline) in consolidation is well supported, the purpose of awake replay (online) is less clear. On one hand, online replay is modulated by environmental novelty (Cheng and Frank, 2008; Foster and Wilson, 2006) as well as changes in reward (Ambrose et al., 2016; Singer and Frank, 2009), and interference with online SWRs impairs the acquisition of spatial tasks (Jadhav et al., 2012), suggestive of a role in learning, if not also consolidation. However, online replay has also been linked with spatial planning and navigation (Foster and Knierim, 2012; Pfeiffer and Foster, 2013; Samsonovich and Ascoli, 2005), consistent with theoretical propositions suggesting replay as a mechanism for exploring potential routes or extracting goal-directed heading vectors (Bush et al., 2015; Erdem and Hasselmo, 2012, 2014; Gupta et al., 2010) and

CrossMark

© 2017 The Authors. Published by Elsevier Inc.
This is an open access article under the CC BY license (http://creativecommons.org/licenses/by/4.0/).

experimental results demonstrating preferential replay of goal-oriented trajectories (Pfeiffer and Foster, 2013).

The range of functions attributed to online replay may relate to apparent distinctions in the forms it can take. For example, the sequence in which place fields are reactivated during replay can recapitulate their order on the track (forward replay) (Diba and Buzsáki, 2007; Lee and Wilson, 2002; Skaggs and McNaughton, 1996) or invert it (reverse replay) (Foster and Wilson, 2006), a dichotomy that also exists for offline replay (Ólafsdóttir et al., 2016; Wikenheiser and Redish, 2013). While forward replay was originally linked with movement initiation and reverse replay with goal arrival (Diba and Buzsáki, 2007), that distinction is now less secure (e.g., Gupta et al., 2010), and it appears reverse replay may be important for reinforcement learning (Ambrose et al., 2016; Foster and Knierim, 2012). Furthermore, although online replay typically encompasses locations close to the animal's current location (Davidson et al., 2009; Dupret et al., 2010; O'Neill et al., 2006; Pfeiffer and Foster, 2013), it can also be remote, representing distant portions of the current environment not related to current goals (Davidson et al., 2009; Gupta et al., 2010), a portion of an environment being avoided (Wu et al., 2017), or even entirely distinct enclosures (Jackson et al., 2006; Karlsson and Frank, 2009).

Thus, although navigation is sometimes associated with forward replay of proximal locations, perhaps supporting goal-directed navigation, equally often online replay is not navigationally relevant, depicting remote locations away from important goals. The factors that govern the switch between these two forms of replay remain unclear. What determines the content of reactivations? Could task demands dictate whether replay is conducted for the purpose of planning or consolidation? If so, does the occurrence of navigationally relevant replay contribute to accurate spatial decisions?

Here we analyze place cell replay occurring during periods of immobility interspersing a self-paced spatial decision task. We find that, after pausing, rats transition rapidly from a state in which they preferentially exhibit replay associated with the ongoing task to a disengaged state, in which replay was directed toward remote sections of the track and also incorporated grid cells from deep layers of the medial entorhinal cortex. Subsequently, reinstatement of movement was prefixed by a switch back to an engaged state, characterized by forward replay of proximate sections of the track without coherent grid activity. Finally, we observed that reactivations occurring before animals made errors on the task did not exhibit engaged-like replay and errors could be predicted on the basis of the replay content. These results suggest that online replay supports both consolidation and spatial planning, providing the first evidence that switching between these states occurs dynamically according to task demands, and, when animals are engaged in a task, that replay content can be important for accurate spatial behavior.

RESULTS

We recorded CA1 place cells (21–72 cells/session; Figure 1C; Figure S1; Table S1) over 1–5 days in eight rats while they performed a spatial decision task (see STAR Methods and Figure 1A). Animals were required to complete laps on an elevated 6-m Z-track. Specifically, rats began each session at the end of arm 1 and ran to the first corner (between arm 1 and arm 2), where they stopped in order to receive a food reward. Following this, they ran to the second corner (between arm 2 and arm 3) and then on to the end of arm 3, being rewarded at the corners and arm ends. They then ran back to the start of arm 1 in a similar fashion (mean number of laps = 22 [SD = 4.82], mean pause duration = 10.71 s [SD = 16.49 s; Figure S2]). Note, only correct turns at the corners would result in food reward at the next stop. Runs going from arm 1 to arm 3 were labeled outbound and runs going back from arm 3 to arm 1 inbound. With experience, animals became increasingly fluent at the task, making fewer wrong turns at the corners (error versus day, $r = -0.61$, $p = 0.0016$; Figure 1B) and taking less time to complete laps (lap duration versus day, $r = -0.46$, $p = 0.0066$).

Corner stops were divided into two temporal sections: engaged, including periods when the animal had just arrived at the corner (<5 s) and just before movement onto the next track was reinstated (<5 s; Figure 1D, ii), and disengaged, the time in between these two periods (Figure 1D, i). We analyzed reactivation events occurring while animals were at the corners. Reactivations were identified based on increases in multi-unit place cell activity (see STAR Methods for details), and they were limited to periods when the animals' speed remained below 3 cm/s. Place cell activity during reactivations was analyzed using a Bayesian decoding approach (Davidson et al., 2009; Ólafsdóttir et al., 2016; Zhang et al., 1998) to calculate the probability of an animal's location on the track given the observed activity (Figure 1D; Figure S3). In the first instance, to understand which sections of the track were represented, we simply summed the probability distribution over each arm, identifying the one that was most strongly reactivated (Figure 1D, i and ii). Note, because place cell activity is highly directional on linear tracks (McNaughton et al., 1983), inbound and outbound runs on each arm were treated separately (mean Pearson correlation between inbound and outbound ratemaps = -0.0032 [SD = 0.28]; Figure S4). Thus, it was possible to identify reactivation of each of the three arms, in either the inbound or outbound direction.

We identified a total of 1,415 engaged and 3,010 disengaged reactivation events. No differences were found between engaged events occurring at the start and end of corner stops (Figure S5). As such, these periods were combined for all subsequent analyses. Engaged events showed a robust bias to reactivate spatial firing congruent with an animal's current direction of travel (62.26% versus chance, $p < 0.0001$, chance derived from shuffling cell IDs; Figures 2A, i and ii, and 2B). Hence, during inbound runs, inbound representations were more likely to be reactivated and vice versa. Disengaged events showed no such bias, being equally likely to activate congruent or incongruent representations (52.19% versus chance, $p = 0.39$, engaged versus disengaged, $p < 0.0001$; Figure 2B). Moreover, engaged events also showed a strong preference to reactivate either of the tracks adjacent to the animal's current position, the local arms, as opposed to the remote arm (80.70% local reactivation versus chance, $p < 0.0001$; Figures 2A, iii and iv, and 2C). Again, this bias was not present in disengaged events, where the proportion of events where the local arms were reactivated relative

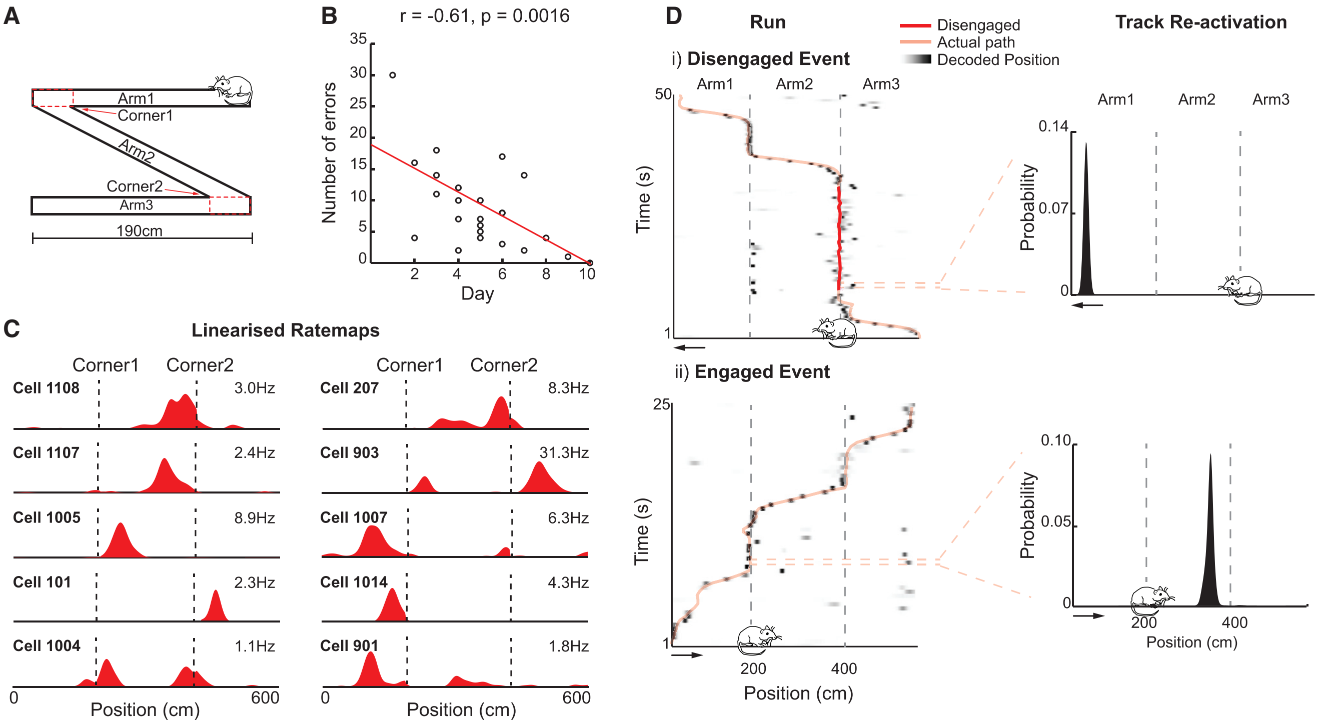

Figure 1. Hippocampal Reactivations during Task Engagement and Disengagement

(A) Rats completed laps on the Z-track.

(B) Number of errors per session versus experimental day with Pearson's r.

(C) Representative linearized outbound place cell ratemaps for 10 CA1 place cells. x axis, track position (cm); y axis, firing rate scaled to each cell's peak rate (shown above the ratemap).

(D) Representative disengaged and engaged reactivation events. (i) Left: an example path (pink) is superimposed on the posterior probability matrix over position (based on periods when animals were running >10 cm/s), with darker shades indicating higher probability. Position of cartoon rat indicates the animal's location and heading direction during the event. Disengaged periods are highlighted in red. x axis, track position (cm); y axis, time (s); bin size, 500 ms. Right: position decoding of a reactivation event is shown (based on periods when animals were stationary, <3 cm/s). x axis, track position (cm); y axis, probability of position. (ii) Same is shown as in (i) but for an engaged reactivation event. See also Figures S1–S4.

to the remote arm was not different to chance (71.42% versus chance, $p = 0.31$; engaged versus disengaged, $p < 0.0001$). Importantly, the results could not be explained by differences in ripple power (Figure S6) or movement speed (Figure S7) during the two types of events. Although disengaged events were characterized by lower theta-band power than engaged events (log[theta power/delta power] during engaged = 0.31 [SD = 0.41], disengaged = 0.23 [SD = 0.39], $p < 0.0001$; Figure S6; log[theta power/delta power] during running = 0.64 [SD = 0.42]), results were unchanged by the exclusion of events with high theta power (i.e., analysis limited to events with theta power <1 SD below mean power during running; Figure S7; see also Figure S8 for a detailed analysis of LFP profile during different task epochs). No difference in ripple power was found between the different event categories (Figure S9). Thus, reactivation events occurring while animals were engaged in the task were more likely to incorporate place cells immediately relevant to that task, representing adjacent sections of the track, and be congruent with the animal's direction of travel.

To better understand the time course governing the transition between the engaged and disengaged states, we examined how the proportion of local and congruent reactivations varied as a function of the time since arrival at, as well as departure from, the corners. Specifically, while animals were stationary (<3 cm/s) at the corners, we calculated the proportion of congruent/local events in 5 s windows (advancing in 2.5 s increments), and we assessed at what time point the proportions were no longer significantly above chance. Following corner arrival, the proportion of congruent events remained above chance for the first 12.5 s (Figure 2D, i). A bias for congruent reactivations was also present for the last 12.5 s preceding corner departure (Figure 2D, ii). The analysis of local reactivations revealed a similar pattern, namely, the proportion of events classified as local remained above chance for the first 10 s after corner arrival (Figure 2E, i) and for the last 10 s prior to exit (Figure 2E, ii). Similarly, ripple power was higher immediately after arrival and just before departure from a corner (Figure S10). Thus, for the first and last 10–15 s of stopping periods, hippocampal reactivations remained in an engaged state, after which they sharply transitioned to a disengaged state. Subsequently, we examined the trajectories encoded during reactivation events.

A Bayesian approach was applied to calculate, for each 10-ms bin within an event, the probability distribution over track position (Zhang et al., 1998). We then applied a line-fitting procedure

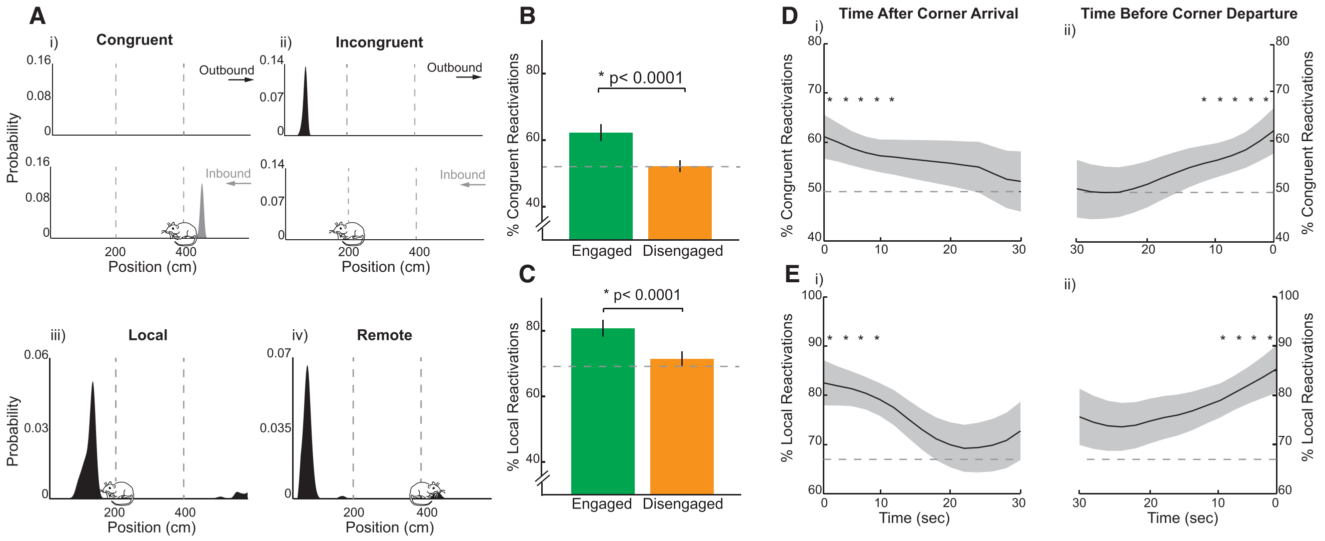

Figure 2. Track Reactivations Are Modulated by Task Engagement

(A) Representative position decoding for a congruent (i), incongruent (ii), local (iii), and remote (iv) reactivation event. Upper panels: decoding was performed separately with ratemaps corresponding to outbound (top) and inbound (bottom) runs. Reactivation events decoding to a run consistent with the animal's current trajectory were categorized as congruent (left) as opposed to incongruent (right). Lower panels: events reactivating positions on the arms immediately ahead or behind the animal were categorized as local (iii), while events reactivating positions on the remote arm were categorized as remote (iv). Position of animal during event is indicated by the location of the cartoon rat. x axis, track position (cm); y axis, probability of position.

(B) Proportion of engaged (green) and disengaged (amber) reactivation events categorized as congruent. Error bars indicate 95% confidence interval based on bootstrapped data.

(C) Same as (B) but for local reactivations.

(D) Proportion of congruent reactivations as a function of time after corner arrival (i) and before corner departure (ii). Shaded areas show 1 SD of bootstrapped data. x axis, time (s); bin size, 2 s; y axis, proportion of congruent reactivations.

(E) Same as (D) but for local reactivations (*p < 0.05 versus chance). See also Figures S5–S10.

(Davidson et al., 2009; Ólafsdóttir et al., 2016) to the resulting posterior probability matrix to identify possible replay trajectories (Figure 3A, i and ii). Again, events were classified as either outbound or inbound depending on which produced the higher best-fit line. Putative events whose rank against their own spatial shuffle distribution exceeded the 97.5th percentile, and which occurred while the animal was stationary (<3 cm/s), were classified as replay events (Figure S11; Table S1).

We identified a total of 149 engaged replay events and 364 disengaged replay events. As before, we saw that engaged replay events preferentially reactivated spatial representations congruent with an animal's current direction of travel (60.82% versus chance, chance derived from shuffling of engaged and disengaged events, p = 0.024; engaged versus disengaged, p = 0.013; Figure 3B). Disengaged events exhibited no bias, being equally likely to represent trajectories moving in either direction (50.50% versus chance, p = 0.89). Furthermore, a clear majority of engaged events replayed trajectories close to the animal (mean position of replay trajectory <60 cm from animal, 70.21% versus chance, p < 0.001, mean distance from animal at start of event = 69.32 cm [SD = 80.78 cm]), whereas disengaged events showed a preference for replaying trajectories remote to the animal (42.03% versus chance, p = 0.0006; engaged versus disengaged, p < 0.0001, mean distance from animal at start of event = 134.48 cm [SD = 119.17 cm], p < 0.0001; Figures 3C, 3F, and 3G). Moreover, we classified replay events as forward or reverse based on the slope of the best-fit line, finding that the majority of engaged replay events were forward events (75.8% versus chance, p < 0.0001), while disengaged periods did not show a preference for forward or reverse replay (53.3% versus chance, p = 0.093, engaged versus disengaged, p < 0.0001; Figure 3D). Importantly, these differences could not be explained by the robustness of the replayed trajectories; although we identified a higher number of disengaged replay events than engaged events, the percentage of putative events qualifying as replay events for the two categories was not different (36.88% versus 38.60%, p = 0.28; Figure 3E).

Finally, we did not find a difference in the proportion of events depicting paths ahead of the animal during engaged and disengaged periods (i.e., prospective replay, engaged 63.09% versus disengaged 68.22%, p = 0.87; Figure 3H). It should be noted that it is unlikely that the observed difference between engaged and disengaged periods result from theta sequences (Foster and Wilson, 2006; Gupta et al., 2012; Johnson and Redish, 2007) or phase precession (O'Keefe and Recce, 1993); theta power during engaged and disengaged replay events did not differ (log[theta/delta] power during engaged = 0.11, disengaged = 0.13, p = 0.65; Figure S12; log[theta power/delta power] during running = 0.64 [SD = 0.42]), and results were unchanged when replay trajectories were required to exceed 2 m in length or when they were limited to events that co-occurred with SWRs (Figure S13). In sum, during periods of task engagement, replay preferentially depicted task-relevant place cell

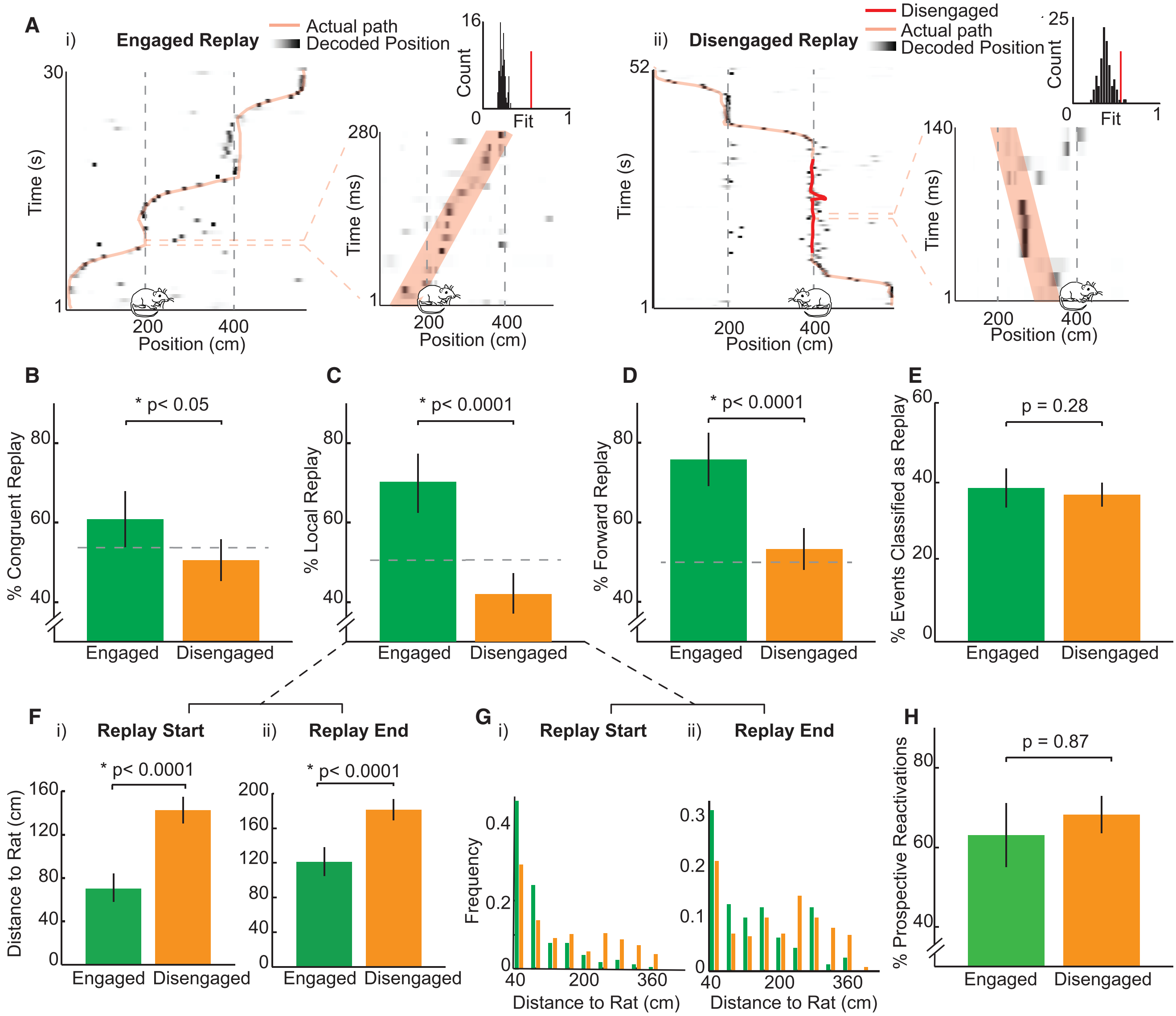

Figure 3. The Content of Replay Trajectories Is Modulated by Task Engagement

(A) Representative engaged (i) and disengaged (ii) replay events. (i) Left: path (pink) is superimposed on position decoding, with darker shades indicating higher probability of position. Cartoon rat indicates animal's position during event. x axis, track position (cm); y axis, time (s); bin size, 500 ms. Right: position decoding during a replay event is shown, with best-fit trajectory superimposed in pink. x axis, track position (cm); y axis, time (ms); bin size, 10 ms. Inset: replay event best-fit trajectory versus shuffle distribution is shown; red bar indicates best-fit trajectory score of original event. (ii) Same is shown as in (i) but for a disengaged event. Disengaged period is highlighted in red.

(B) Proportion of engaged (green) and disengaged (amber) replay events categorized as congruent. Error bars show 95% confidence interval based on bootstrapped data.

(C and D) Same as (B) but for local (C) and forward (D) replay.

(E) Proportion of putative engaged (green) and disengaged (amber) events categorized as replay events.

(F) Distance between animal's actual position and start (i) or end (ii) of the replay trajectory.

(G) Probability distribution of distances between animal and start (i) or end (ii) of the replay trajectory.

(H) Proportion of engaged (green) and disengaged (amber) replay events categorized as prospective (i.e., depicting trajectories ahead of the animal). See also Figures S11–S13.

sequences: trajectories congruent with an animal's current direction of travel, that were close to its current position, and that maintained the order in which cells were activated during experience. Conversely, during disengaged periods, replay trajectories were less biased; they were equally likely to depict motion in either direction regardless of the animal's heading, showed no preference for forward and reverse sequences, and were more focused on distant regions of the track.

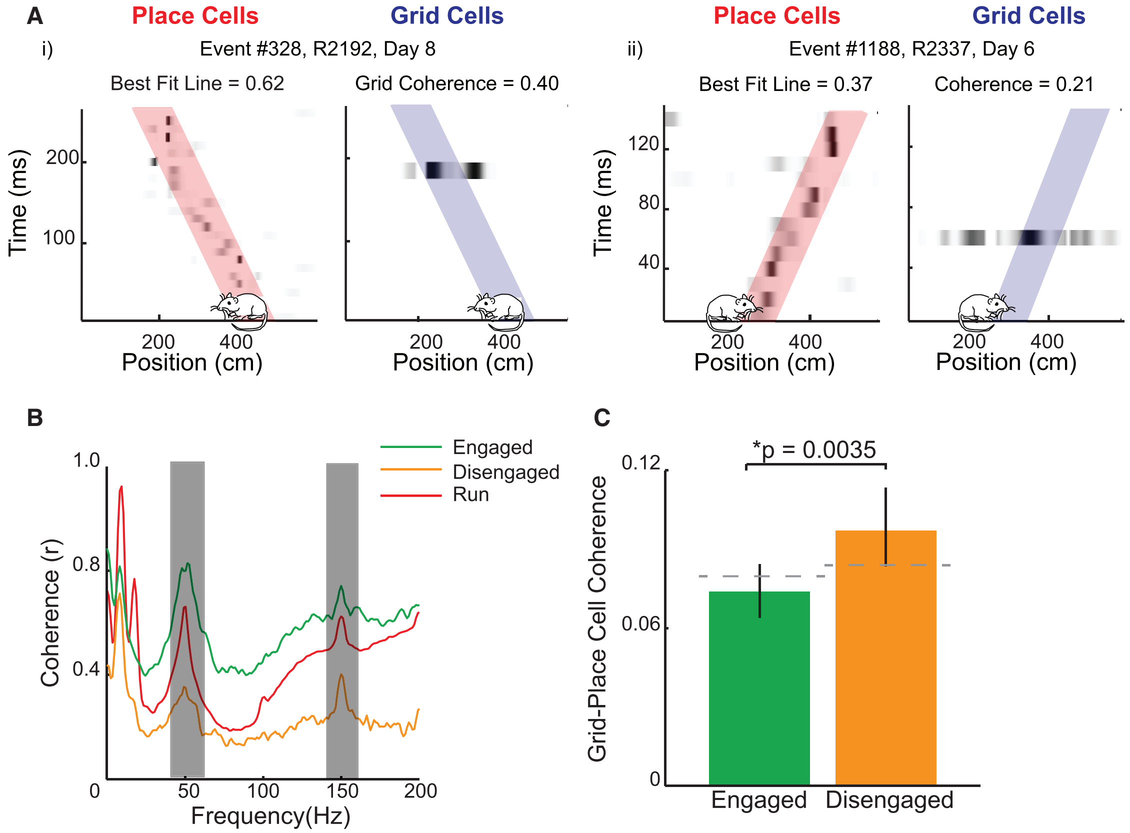

Figure 4. Grid-Place Cell Coherence Is Accentuated during Periods of Task Disengagement

(A) Representative replay events incorporating at least one active grid cell. (i) Left: position decoding is based on place cell activity during replay event, with darker shades indicating higher probability of position and best-fit trajectory superimposed in pink. Title indicates best-fit score. Right: Same is shown as at the left but for grid cell activity, with best-fit trajectory from concurrent place cell activity superimposed in blue. Title indicates coherence between place and grid cells during event. x axis, position on track (cm); y axis, time (ms). Position and head direction of cartoon rat indicate animal's location and direction of travel during the event. (ii) Same is shown as in (i) but for a replay event recorded from a different animal.

(B) Coherence between hippocampal and MEC LFP during engaged (green) and disengaged (amber) corner periods. For reference, the coherence observed during running (>10 cm/s) is shown in red. x axis, LFP frequency (Hz); y axis, LFP coherence (r). Grey area covers portion of the LFP contaminated by mains noise.

(C) Mean grid-place cell coherence during hippocampal replay for engaged (green) and disengaged (amber) periods. Horizontal dashed line shows coherence expected by chance. See also Figure S14.

Medial entorhinal cortex (MEC) grid cells are also known to exhibit replay (O'Neill et al., 2017; Ólafsdóttir et al., 2016). During rest, grid cells from the deep layers of the MEC, the primary cortical projection target of the hippocampus, replay coherently with place cells but do so with a slight delay (11 ms) (Ólafsdóttir et al., 2016). Conversely, grid cells from the superficial layers of the MEC, upstream of the hippocampus, replay trajectories independently, an effect that is more pronounced during task engagement than rest (O'Neill et al., 2017). Plausibly, the transmission of replayed trajectories from CA1 to the deep layers of the MEC during quiescence might support consolidation, whereas hippocampal-independent replay in superficial MEC is more likely to be linked with navigational planning (Bush et al., 2015) and need not engage deep MEC. To investigate this distinction, we analyzed place cell replay that co-occurred with at least one grid cell spike (Figure 4A, i and ii) recorded from deep MEC (layers V/VI) in seven of the eight rats included in the study. During both engaged and disengaged periods, we observed LFP coherence between the hippocampus and MEC in the theta (6- to 12-Hz) and ripple (150- to 250-Hz) bands (Figure 4B; Figure S14 for an alternative LFP coherence analysis between hippocampus and MEC). Specifically, we found grid cells were coordinated with place cell replay during disengaged periods (grid-place coordination = 0.097 [SD = 0.08] versus chance, p = 0.011), but not engaged periods (grid-place coordination = 0.074 [SD = 0.037] versus chance, p = 0.75), with the amount of coherence between the two periods differing significantly (engaged versus disengaged, p = 0.0035; Figure 4C). As such, these results suggest a further functional distinction between replay occurring during engaged and disengaged periods; with the latter, engagement of cortical grid cells appear to provide a mechanism capable of supporting system-level consolidation.

To what extent is the content of engaged reactivations important for accurate task performance? To address this question, we analyzed events preceding correct and incorrect turns separately (Figure 5A, i and ii). Due to the relatively small number of

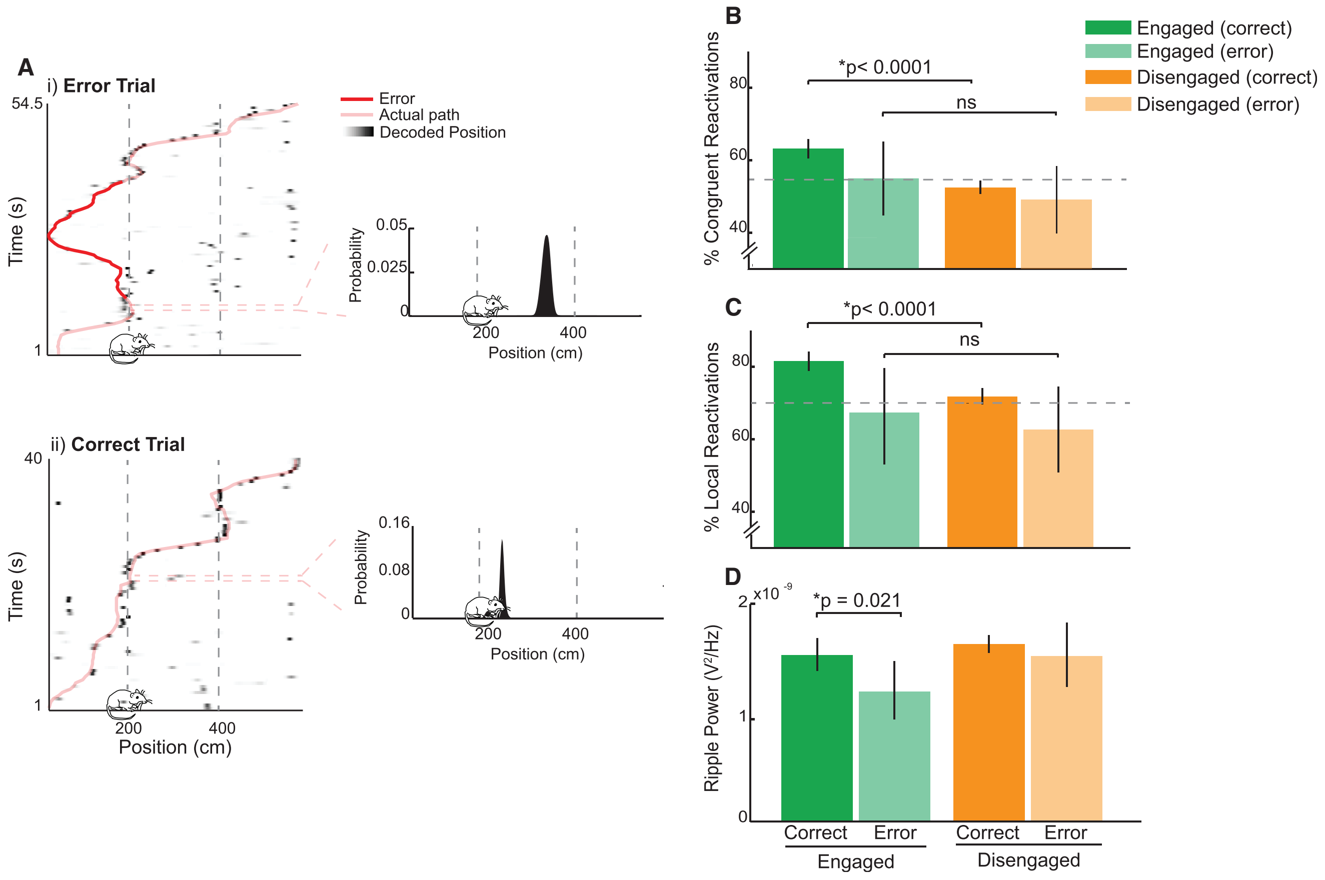

Figure 5. Effect of Task Engagement on Reactivations Is Only Observed before Accurate Turns

(A) Representative reactivation events preceding incorrect and correct spatial choices. (i) Left: rat's path (pink) is superimposed on position probability matrix, with darker shades indicating higher probability of position. Erroneous portion of the path is shown in red. x axis, position on the track (cm); y axis, time (s); bin size, 500 ms. Right: position decoding of reactivation event is shown. x axis, position on the track (cm); y axis, probability of position. Rat location is indicated by the position of the cartoon rat. x axis, track position (cm); y axis, probability of position. (ii) Same is shown as in (i) but for a reactivation event preceding a correct spatial choice.

(B) Proportion of engaged (green) and disengaged (amber) events classified as congruent. Darker bars show proportions for events preceding a correct spatial choice, lighter bars indicate errors. Error bars indicate 95% confidence interval based on bootstrapped data. Interaction between error/correct and engaged/disengaged was not significant ($p = 0.19$, difference engaged correct versus engaged error = 9.7%, difference disengaged error versus disengaged correct = 3.4%). *ns refers to multiple pairwise comparisons for congruent reactivations; error engaged versus correct disengaged, $p = 0.46$; error engaged versus error disengaged, $p = 0.21$; correct disengaged versus error disengaged, $p = 0.14$.

(C and D) Same as (B) but for local reactivations (C) and ripple power (D) during reactivations. Again, interaction was not significant for either analysis (local reactivations, $p = 0.14$; difference engaged correct versus engaged error = 15.0%, difference disengaged error versus disengaged correct = 7.6%; ripple power, $p = 0.16$; difference engaged correct versus engaged error = 3.46×10^{-10}, difference disengaged error versus disengaged correct = 1.25×10^{-10}). *ns refers to multiple pairwise comparisons for local reactivations; error engaged versus correct disengaged, $p = 0.77$; error engaged versus error disengaged, $p = 0.31$; correct disengaged versus error disengaged, $p = 0.062$.

errors (mean wrong turns per session = 8.88 [SD = 6.88]), analyses were limited to simple reactivation events. For events preceding correct turns, we saw, as before, a clear distinction between engaged and disengaged reactivations (Figures 5B and 5C). Thus, engaged reactivations had a tendency to be congruent with, and local to, an animal's position (62.79% congruent versus chance, $p < 0.0001$; 81.5% local versus chance, $p < 0.0001$), while disengaged reactivations exhibited no such bias (52.34% congruent versus chance, $p = 0.19$; 71.76% local versus chance, $p = 0.12$; engaged versus disengaged, both p values < 0.0001). However, for events preceding incorrect turns, this distinction was absent. As expected, disengaged events exhibited no bias toward congruent or local reactivations (48.76% congruent versus chance, $p = 0.94$; 62.71% local versus chance, $p = 0.89$), but neither did, in this instance, engaged events (54.44% engaged, $p = 0.76$ versus chance; 67.35% local versus chance, $p = 0.67$; engaged versus disengaged, both p values > 0.20; Figures 5B and 5C). Indeed, the proportion of engaged local events preceding incorrect turns was significantly lower than that preceding correct turns ($p < 0.0001$; Figure 5C); a marginally significant effect in the same direction was observed for congruent events

(p = 0.062; Figure 5B). However, due to the small number of error events (engaged error events = 90, disengaged error events = 121), we did not find a significant interaction between levels of task engagement and the accuracy of future decisions (based on bootstrapping difference scores for events preceding correct and erroneous turns, congruent analysis p = 0.19, local analysis p = 0.14; Figures 5B and 5C).

To understand if the observed difference was also manifest at the network level, we examined how power in the ripple band of the LFP (150–250 Hz) varied between events preceding correct and incorrect choices. Ripple power did not differ between disengaged events preceding correct and incorrect turns (correct = 1.73×10^{-9} V^2/Hz, error = 1.61×10^{-9} V^2/Hz, p = 0.76; Figure 5D). However, for engaged events alone, we saw that ripple power was reduced prior to errors (correct = 1.62×10^{-9} V^2/Hz, error = 1.27×10^{-9} V^2/Hz, p = 0.021; Figure 5D). Again, we found the interaction analysis was in the right direction but did not reach statistical significance (p = 0.16). Thus, the content of engaged reactivations predicts the accuracy of future decisions; prior to making erroneous spatial decisions, reactivations were less task focused and were accompanied by a reduction in ripple power.

Finally, to corroborate the suggestion that task-relevant reactivations are important for accurate spatial behavior, we trained a binary classification decision tree (Kohavi and Quinlan, 2002; Rokach and Maimon, 2008) to predict the occurrence of errors based on all three of the measures we had obtained for the preceding reactivations (see STAR Methods). Specifically, the decision tree was trained with data specifying for each event if it was local to the animal, congruent with the current direction of travel, as well as its mean power in the ripple band. Training and test data were partitioned using a 10-fold cross-validation scheme; on each of 10 iterations, the model was fit to 90% of the data and tested on the remaining 10%, using different portions of the data on each fold. Trees trained on reactivations occurring during engaged periods predicted the outcome of subsequent decisions correctly 62.8% of the time (p = 0.001 compared to 1,000 iterations of the model fit to shuffled data, mean shuffle prediction accuracy = 49.6%). Similarly, training using just the locality and congruence of events also allowed for better-than-chance prediction performance (60.3% accuracy, p = 0.02 versus shuffle). Note, it is likely that the classification accuracy is not higher, precisely because reactivations occurring before errors are heterogeneous. In contrast to the engaged periods, disengaged reactivations were uninformative about the accuracy of subsequent decisions (55.2% decision accuracy, p = 0.09 compared to 1,000 iterations of the model fit to shuffled data, mean shuffle prediction accuracy = 49.8%). Thus, the content of reactivations during periods of task engagement predicts whether the animal's future choice will be accurate.

DISCUSSION

We have described findings showing a clear distinction between replay occurring during periods in which animals were engaged in a spatial task and periods in which they were simply resting on the track. When animals were preparing to make a spatial decision and initiate movement, or when they had just completed a trajectory, replay tended to be focused on the immediate environment, reactivating trajectories consistent with the animal's position and heading, in a forward direction. Conversely, during prolonged stops at the corners of the track, replay trajectories were distributed across the apparatus, were equally likely to propagate in a forward or reverse direction, and incorporated entorhinal grid cells from the deep layers of the MEC. The transition between these states occurred relatively rapidly, being task focused for 10–15 s before and after movement, and the presence of task-focused reactivations during periods when animals were engaged with the task was found to predict the accuracy of their subsequent decisions. In sum, these results show that the content of awake hippocampal reactivations can transition predictably and dynamically in accordance with task demands and that the content of hippocampal replay may determine the accuracy of spatial behavior.

Replay, particularly online replay, has been noted to support a range of neurobiological phenomena, including memory consolidation (Girardeau et al., 2009; Wilson and McNaughton, 1994), mental exploration (Derdikman and Moser, 2010; Gupta et al., 2010; Ólafsdóttir et al., 2015), spatial planning (Pfeiffer and Foster, 2013), decision making (Singer et al., 2013), and reinforcement learning (Foster and Knierim, 2012; Foster and Wilson, 2006). Indeed, each of these proposed roles enjoys some degree of theoretical and empirical support. However, the extent to which these functions correspond to different types of replay is unclear. Our results suggest current task demands may dictate the operational mode of replay and, thus, its content. Namely, during periods of task engagement, when an animal might be expected to be planning or perhaps rehearsing future choices, replay favors adjacent positions and preserves the normal ordering of place cells, consistent with previous work linking replay at decision points with behavior (Diba and Buzsáki, 2007; Pfeiffer and Foster, 2013; Singer et al., 2013). In contrast, when animals are disengaged from the ongoing task, replay is less focused, reactivating a heterogeneous mixture of positions and paths (Davidson et al., 2009; Gupta et al., 2010). Plausibly, this latter state, which reflects a composite of recent experiences and engages downstream cortical targets, is likely to be linked with consolidation, a view consistent with previous work (Girardeau et al., 2009). Furthermore, we showed that successful navigation is predicted specifically by the occurrence of replay focused on the current task. Previous studies have shown performance on spatial memory tasks correlates with the content of replay (Dupret et al., 2010; Papale et al., 2016; Singer et al., 2013), yet we are the first to show that only when an animal is actively engaged in a task does the content of replay predict the accuracy of future spatial decisions. While a causal link is still to be proven, this predictive relationship strengthens the case that different forms of replay perform different functions, indicating that forward local replay is especially important for spatial planning.

In contrast, it is less clear what function the heterogeneous replay occurring during prolonged stationary periods might support. In humans, periods of task disengagement are known to be accompanied by activity in a default mode network (Buckner et al., 2008), which can often incorporate the hippocampus and is characterized by recall of previous events and

autobiographical memories (Greicius et al., 2009; Gupta et al., 2010; Spreng et al., 2009). As such, it is tempting to speculate that replay during these periods has a role to play in learning from past experience, potentially being important for memory consolidation (Girardeau et al., 2009), or could reflect mental exploration of the task environment (Derdikman and Moser, 2010; Gupta et al., 2010).

Finally, we also explored the dynamics of the transition between engaged and disengaged periods, showing that it was rapid but by no means immediate upon corner arrival and departure. How might this switch be mediated? The prefronal cortex (PFC) might possibly play an important role, given its interaction with the hippocampus is known to be modulated by task demands (Place et al., 2016). Another candidate region is the MEC. Recent experimental work has shown that replay can originate independently in the MEC and hippocampus (O'Neill et al., 2017). In turn, replay of MEC grid cells has been suggested to play a role in navigation (Bush et al., 2015; Erdem and Hasselmo, 2012, 2014; Kubie and Fenton, 2012), while replay originating from the hippocampus seems more likely to contribute to mnemonic processes. As such, we propose that, during engaged periods, task-focused replay is initiated in the MEC but ultimately incorporates place cells. Conversely, during quiescence, replay is mainly hippocampal initiated, and as such it reflects the diverse range of trajectories that animals have recently experienced.

STAR★METHODS

Detailed methods are provided in the online version of this paper and include the following:

- KEY RESOURCES TABLE
- CONTACT FOR REAGENT AND RESOURCE SHARING
- EXPERIMENTAL MODEL AND SUBJECT DETAILS
- METHOD DETAILS
  - Recording
  - Experimental apparatus and protocol
  - Behavioral task performance
  - Data inclusion/exclusion
  - Data analysis
  - Reactivation analysis
  - Grid coherence analysis
  - Local field potential analysis
  - Model based prediction
  - Histology
- QUANTIFICATION AND STATISTICAL ANALYSIS
- DATA AND SOFTWARE AVAILABILITY

SUPPLEMENTAL INFORMATION

Supplemental Information includes fourteen figures and one table and can be found with this article online at https://doi.org/10.1016/j.neuron.2017.09.035.

AUTHOR CONTRIBUTIONS

H.F.O. and C.B. conceived of the project jointly. H.F.O. and F.C. performed surgeries. H.F.O. carried out experiments. H.F.O. and C.B. performed the analyses. All authors wrote the manuscript.

ACKNOWLEDGMENTS

This work was supported by the Wellcome Trust and the Royal Society. The authors thank Robin Hayman, Francesca Cacucci, and Daniel Bush for comments on the manuscript.

Received: March 30, 2017
Revised: July 19, 2017
Accepted: September 22, 2017
Published: October 19, 2017

REFERENCES

Ambrose, R.E., Pfeiffer, B.E., and Foster, D.J. (2016). Reverse replay of hippocampal place cells is uniquely modulated by changing reward. Neuron *91*, 1124–1136.

Barry, C., Hayman, R., Burgess, N., and Jeffery, K.J. (2007). Experience-dependent rescaling of entorhinal grids. Nat. Neurosci. *10*, 682–684.

Battaglia, F.P., Sutherland, G.R., and McNaughton, B.L. (2004). Hippocampal sharp wave bursts coincide with neocortical "up-state" transitions. Learn. Mem. *11*, 697–704.

Buckner, R.L., Andrews-Hanna, J.R., and Schacter, D.L. (2008). The brain's default network: anatomy, function, and relevance to disease. Ann. N Y Acad. Sci. *1124*, 1–38.

Bush, D., Barry, C., Manson, D., and Burgess, N. (2015). Using grid cells for navigation. Neuron *87*, 507–520.

Buzsáki, G. (2015). Hippocampal sharp wave-ripple: a cognitive biomarker for episodic memory and planning. Hippocampus *25*, 1073–1188.

Buzsáki, G., Horváth, Z., Urioste, R., Hetke, J., and Wise, K. (1992). High-frequency network oscillation in the hippocampus. Science *256*, 1025–1027.

Cheng, S., and Frank, L.M. (2008). New experiences enhance coordinated neural activity in the hippocampus. Neuron *57*, 303–313.

Chrobak, J.J., and Buzsáki, G. (1994). Selective activation of deep layer (V-VI) retrohippocampal cortical neurons during hippocampal sharp waves in the behaving rat. J. Neurosci. *14*, 6160–6170.

Chrobak, J.J., and Buzsáki, G. (1996). High-frequency oscillations in the output networks of the hippocampal-entorhinal axis of the freely behaving rat. J. Neurosci. *16*, 3056–3066.

Davidson, T.J., Kloosterman, F., and Wilson, M.A. (2009). Hippocampal replay of extended experience. Neuron *63*, 497–507.

de Lavilléon, G., Lacroix, M.M., Rondi-Reig, L., and Benchenane, K. (2015). Explicit memory creation during sleep demonstrates a causal role of place cells in navigation. Nat. Neurosci. *18*, 493–495.

Derdikman, D., and Moser, M.B. (2010). A dual role for hippocampal replay. Neuron *65*, 582–584.

Diba, K., and Buzsáki, G. (2007). Forward and reverse hippocampal place-cell sequences during ripples. Nat. Neurosci. *10*, 1241–1242.

Dupret, D., O'Neill, J., Pleydell-Bouverie, B., and Csicsvari, J. (2010). The reorganization and reactivation of hippocampal maps predict spatial memory performance. Nat. Neurosci. *13*, 995–1002.

Ego-Stengel, V., and Wilson, M.A. (2010). Disruption of ripple-associated hippocampal activity during rest impairs spatial learning in the rat. Hippocampus *20*, 1–10.

Erdem, U.M., and Hasselmo, M. (2012). A goal-directed spatial navigation model using forward trajectory planning based on grid cells. Eur. J. Neurosci. *35*, 916–931.

Erdem, U.M., and Hasselmo, M.E. (2014). A biologically inspired hierarchical goal directed navigation model. J. Physiol. Paris *108*, 28–37.

Eschenko, O., Ramadan, W., Mölle, M., Born, J., and Sara, S.J. (2008). Sustained increase in hippocampal sharp-wave ripple activity during slow-wave sleep after learning. Learn. Mem. *15*, 222–228.

Foster, D.J., and Knierim, J.J. (2012). Sequence learning and the role of the hippocampus in rodent navigation. Curr. Opin. Neurobiol. *22*, 294–300.

Foster, D.J., and Wilson, M.A. (2006). Reverse replay of behavioural sequences in hippocampal place cells during the awake state. Nature *440*, 680–683.

Girardeau, G., Benchenane, K., Wiener, S.I., Buzsáki, G., and Zugaro, M.B. (2009). Selective suppression of hippocampal ripples impairs spatial memory. Nat. Neurosci. *12*, 1222–1223.

Greicius, M.D., Supekar, K., Menon, V., and Dougherty, R.F. (2009). Resting-state functional connectivity reflects structural connectivity in the default mode network. Cereb. Cortex *19*, 72–78.

Gupta, A.S., van der Meer, M.A., Touretzky, D.S., and Redish, A.D. (2010). Hippocampal replay is not a simple function of experience. Neuron *65*, 695–705.

Gupta, A.S., van der Meer, M.A., Touretzky, D.S., and Redish, A.D. (2012). Segmentation of spatial experience by hippocampal θ sequences. Nat. Neurosci. *15*, 1032–1039.

Jackson, J.C., Johnson, A., and Redish, A.D. (2006). Hippocampal sharp waves and reactivation during awake states depend on repeated sequential experience. J. Neurosci. *26*, 12415–12426.

Jadhav, S.P., Kemere, C., German, P.W., and Frank, L.M. (2012). Awake hippocampal sharp-wave ripples support spatial memory. Science *336*, 1454–1458.

Ji, D., and Wilson, M.A. (2007). Coordinated memory replay in the visual cortex and hippocampus during sleep. Nat. Neurosci. *10*, 100–107.

Johnson, A., and Redish, A.D. (2007). Neural ensembles in CA3 transiently encode paths forward of the animal at a decision point. J. Neurosci. *27*, 12176–12189.

Johnson, L.A., Euston, D.R., Tatsuno, M., and McNaughton, B.L. (2010). Stored-trace reactivation in rat prefrontal cortex is correlated with down-to-up state fluctuation density. J. Neurosci. *30*, 2650–2661.

Karlsson, M.P., and Frank, L.M. (2009). Awake replay of remote experiences in the hippocampus. Nat. Neurosci. *12*, 913–918.

Kohavi, R., and Quinlan, J.R. (2002). Data mining tasks and methods: Classification: decision-tree discovery (New York: Oxford University Press).

Kubie, J.L., and Fenton, A.A. (2012). Linear look-ahead in conjunctive cells: an entorhinal mechanism for vector-based navigation. Front. Neural Circuits *6*, 20.

Lee, A.K., and Wilson, M.A. (2002). Memory of sequential experience in the hippocampus during slow wave sleep. Neuron *36*, 1183–1194.

Maingret, N., Girardeau, G., Todorova, R., Goutierre, M., and Zugaro, M. (2016). Hippocampo-cortical coupling mediates memory consolidation during sleep. Nat. Neurosci. *19*, 959–964.

Marr, D. (1971). Simple memory: a theory for archicortex. Philos. Trans. R. Soc. Lond. B Biol. Sci. *262*, 23–81.

Masimore, B., Kakalios, J., and Redish, A.D. (2004). Measuring fundamental frequencies in local field potentials. J. Neurosci. Methods *138*, 97–105.

McNaughton, B.L., Barnes, C.A., and O'Keefe, J. (1983). The contributions of position, direction, and velocity to single unit activity in the hippocampus of freely-moving rats. Exp. Brain Res. *52*, 41–49.

Mednick, S.C., McDevitt, E.A., Walsh, J.K., Wamsley, E., Paulus, M., Kanady, J.C., and Drummond, S.P. (2013). The critical role of sleep spindles in hippocampal-dependent memory: a pharmacology study. J. Neurosci. *33*, 4494–4504.

O'Keefe, J., and Dostrovsky, J. (1971). The hippocampus as a spatial map. Preliminary evidence from unit activity in the freely-moving rat. Brain Res. *34*, 171–175.

O'Keefe, J., and Nadel, L. (1978). The Hippocampus as a Cognitive Map (Oxford: Clarendon).

O'Keefe, J., and Recce, M.L. (1993). Phase relationship between hippocampal place units and the EEG theta rhythm. Hippocampus *3*, 317–330.

O'Neill, J., Senior, T., and Csicsvari, J. (2006). Place-selective firing of CA1 pyramidal cells during sharp wave/ripple network patterns in exploratory behavior. Neuron *49*, 143–155.

O'Neill, J., Senior, T.J., Allen, K., Huxter, J.R., and Csicsvari, J. (2008). Reactivation of experience-dependent cell assembly patterns in the hippocampus. Nat. Neurosci. *11*, 209–215.

O'Neill, J., Boccara, C.N., Stella, F., Schoenenberger, P., and Csicsvari, J. (2017). Superficial layers of the medial entorhinal cortex replay independently of the hippocampus. Science *355*, 184–188.

Ólafsdóttir, H.F., Barry, C., Saleem, A.B., Hassabis, D., and Spiers, H.J. (2015). Hippocampal place cells construct reward related sequences through unexplored space. eLife *4*, e06063.

Ólafsdóttir, H.F., Carpenter, F., and Barry, C. (2016). Coordinated grid and place cell replay during rest. Nat. Neurosci. *19*, 792–794.

Papale, A.E., Zielinski, M.C., Frank, L.M., Jadhav, S.P., and Redish, A.D. (2016). Interplay between hippocampal sharp-wave-ripple events and vicarious trial and error behaviors in decision making. Neuron *92*, 975–982.

Peyrache, A., Battaglia, F.P., and Destexhe, A. (2011). Inhibition recruitment in prefrontal cortex during sleep spindles and gating of hippocampal inputs. Proc. Natl. Acad. Sci. USA *108*, 17207–17212.

Pfeiffer, B.E., and Foster, D.J. (2013). Hippocampal place-cell sequences depict future paths to remembered goals. Nature *497*, 74–79.

Place, R., Farovik, A., Brockmann, M., and Eichenbaum, H. (2016). Bidirectional prefrontal-hippocampal interactions support context-guided memory. Nat. Neurosci. *19*, 992–994.

Rasch, B., Büchel, C., Gais, S., and Born, J. (2007). Odor cues during slow-wave sleep prompt declarative memory consolidation. Science *315*, 1426–1429.

Rokach, L., and Maimon, O. (2008). Data mining with decision trees: theory and applications (Singapore: World Scientific Publishing Co.).

Rothschild, G., Eban, E., and Frank, L.M. (2017). A cortical-hippocampal-cortical loop of information processing during memory consolidation. Nat. Neurosci. *20*, 251–259.

Samsonovich, A.V., and Ascoli, G.A. (2005). A simple neural network model of the hippocampus suggesting its pathfinding role in episodic memory retrieval. Learn. Mem. *12*, 193–208.

Scoville, W.B., and Milner, B. (1957). Loss of recent memory after bilateral hippocampal lesions. J. Neurol. Neurosurg. Psychiatry *20*, 11–21.

Silva, D., Feng, T., and Foster, D.J. (2015). Trajectory events across hippocampal place cells require previous experience. Nat. Neurosci. *18*, 1772–1779.

Singer, A.C., and Frank, L.M. (2009). Rewarded outcomes enhance reactivation of experience in the hippocampus. Neuron *64*, 910–921.

Singer, A.C., Carr, M.F., Karlsson, M.P., and Frank, L.M. (2013). Hippocampal SWR activity predicts correct decisions during the initial learning of an alternation task. Neuron *77*, 1163–1173.

Sirota, A., Csicsvari, J., Buhl, D., and Buzsáki, G. (2003). Communication between neocortex and hippocampus during sleep in rodents. Proc. Natl. Acad. Sci. USA *100*, 2065–2069.

Skaggs, W.E., and McNaughton, B.L. (1996). Replay of neuronal firing sequences in rat hippocampus during sleep following spatial experience. Science *271*, 1870–1873.

Spreng, R.N., Mar, R.A., and Kim, A.S. (2009). The common neural basis of autobiographical memory, prospection, navigation, theory of mind, and the default mode: a quantitative meta-analysis. J. Cogn. Neurosci. *21*, 489–510.

Suzuki, S.S., and Smith, G.K. (1985). Single-cell activity and synchronous bursting in the rat hippocampus during waking behavior and sleep. Exp. Neurol. *89*, 71–89.

van de Ven, G.M., Trouche, S., McNamara, C.G., Allen, K., and Dupret, D. (2016). hippocampal offline reactivation consolidates recently formed cell assembly patterns during sharp wave-ripples. Neuron *92*, 968–974.

Vanderwolf, C.H. (1969). Hippocampal electrical activity and voluntary movement in the rat. Electroencephalogr. Clin. Neurophysiol. *26*, 407–418.

Wierzynski, C.M., Lubenov, E.V., Gu, M., and Siapas, A.G. (2009). State-dependent spike-timing relationships between hippocampal and prefrontal circuits during sleep. Neuron *61*, 587–596.

Wikenheiser, A.M., and Redish, A.D. (2013). The balance of forward and backward hippocampal sequences shifts across behavioral states. Hippocampus *23*, 22–29.

Wilson, M.A., and McNaughton, B.L. (1994). Reactivation of hippocampal ensemble memories during sleep. Science *265*, 676–679.

Wu, C.T., Haggerty, D., Kemere, C., and Ji, D. (2017). Hippocampal awake replay in fear memory retrieval. Nat. Neurosci. *20*, 571–580.

Zhang, K., Ginzburg, I., McNaughton, B.L., and Sejnowski, T.J. (1998). Interpreting neuronal population activity by reconstruction: unified framework with application to hippocampal place cells. J. Neurophysiol. *79*, 1017–1044.

STAR★METHODS

KEY RESOURCES TABLE

REAGENT or RESOURCE	SOURCE	IDENTIFIER
Experimental Models: Organisms/Strains		
Lister Hooded rats	Charles River	N/A, http://www.criver.com/products-services/basic-research/find-a-model/lister-hooded?loc=GB
Software and Algorithms		
MATLAB	Mathworks. MA	RRID: SCR_001622, https://uk.mathworks.com/products/matlab.html
Chemicals		
Cresyl violet	Sigma Aldrich	Product code: C5042, http://www.sigmaaldrich.com/catalog/product/sigma/c5042?lang=en&region=US
Histoclear	National Diagnostics	Product code: HS-202, https://www.nationaldiagnostics.com/histology/product/histo-clear-ii
Other		
Tint Spike sorting software	Axona	Product code: COMP/TINT01, http://axona.com/products
Recording system (pre-amp & systems unit)	Axona	Product code: Dacq/USB64, http://axona.com/products
Omnetic connectors (microdrive assembly)	Genalog	Product code: A79026-001,http://genalog.com/genalog-linecard/omnetics/
Single-screw mouse microdrive	Axona	Product code: MDMR-01M1, http://axona.com/products
4x16channel headstage preamplifiers	Axona	Product code: HS-116M1D, http://axona.com/products
Microwire (17um, platinum iridium)	California Fine Wire Company	Product code:100167, http://www.calfinewire.com/datasheets/100167-platinum10iridium.html
NanoZ plating equipment	Multichannel Systems	nanoZ, http://www.multichannelsystems.com/products/nanoz
4xfine wire tethers	Axona	Product code: HS16-CAB3, http://axona.com/products

CONTACT FOR REAGENT AND RESOURCE SHARING

Further information and requests for resources should be directed to and will be fulfilled by the Lead Contact H. Freyja Olafsdottir (h.olafsdottir@ucl.ac.uk)

EXPERIMENTAL MODEL AND SUBJECT DETAILS

Eight male Lister Hooded rats were used in this study. All procedures were approved by the UK Home Office, subject to the restrictions and provisions contained in the Animals Scientific Procedures Act of 1986. All rats (330-400 g/13-18weeks old at implantation) received two single-screw microdrives (Axona Ltd.) fixed to a pair of 2x16channels omnetics connectors (Genalog Ltd.), each carrying eight tetrodes of twisted 17μm HM-L coated platinum iridium wire (90% and 10%, respectively; California Fine Wire), targeted to the right CA1 (ML: 2.2mm, AP: 3.8mm posterior to Bregma) and left medial entorhinal cortex (MEC) (ML = 4.5mm, AP = 0.3-0.7 anterior to the transverse sinus, angled between 8-10°). Wires were platinum plated to reduce impedance to 200-300kΩ at 1kHz (NanoZ). After rats had recovered from surgery they were maintained at 90% of free-feeding weight with ad libitum access to water, and were housed individually on a 12 hr light/dark cycle.

METHOD DETAILS

Recording

Screening was performed post-surgically after a 1-week recovery period. An Axona recording system (Axona Ltd., St Albans, UK) was used to acquire positional data and the single-units, via 4x16channel headstage preamplifiers connected to the recording system using 4 fine wire tethers (Axona Ltd., for details of the recording system and basic recording protocol see Barry et al. (2007)). The position and head direction of the animals was inferred using an overhead video camera to record the location of two light-emitting

diodes (LEDs) mounted on the animals' head-stages (50Hz). Tetrodes were gradually advanced in 62.5um steps across days until place cells (CA1) and grid cells (MEC) were identified.

Experimental apparatus and protocol

The experiment was run during the animals' light period to encourage quiet restfulness during the rest session. Animals ran on a Z-shaped track, elevated 75cm off the ground with 10cm wide runways. The two parallel tracks of the Z (190cm each) were connected by a diagonal section (220cm). The entire track was surrounded by plain black curtains with no distal cues. During each track session, animals were required to complete laps on the elevated Z-track. Specifically, the animals were required to run from the start of Arm1 to the end of Arm3, stopping at the track corners and ends in order to receive a food reward. If the animals made a wrong turn at the corners, reward was withheld. Four animals (R2142, R2192, R2198, and R2217) were trained to run on the track for 3 days before recording commenced. For the other animals (R2242, R2335, R2336, R2337), recordings were made from the first day of exposure to the Z-track task.

Following the track session, rats were placed in the rest enclosure for 90 min. The rest enclosure consisted of a cylindrically shaped environment (18cm diameter, 61cm high) with a towel placed at the bottom and was located outside of the curtains which surrounded the Z-track. Animals were not able to see the surrounding room while in the rest enclosure. Prior to the experiment, rats had been familiarised with the rest enclosure for at least 7 days. Animals R2242, R2335, R2336 and R2337, were also placed in the rest enclosure for 90 min prior to the first Z-track session on day 1 of the experiment. Recordings from this 'pre-rest' session were not analyzed as part of this study. Following the rest session, animals completed a 20min foraging session in an open field environment. This session was included to enable functional classification of MEC cells and was not analyzed in the current study.

Behavioral task performance

To index behavioral fluency we used two measures. First, we recorded the number of incorrect turns animals made at the corners of the track in each session. Second, we calculated the mean time animals took to complete each lap (total session duration / number of laps). Learning across days was assessed by correlating these behavioral measures with the number of days of experience the animal had had on the track.

Data inclusion/exclusion

Sessions where the median decoding error during track running (see 'Data Analysis' section below for details) did not exceed 30cm (5% of the length of the track) were included in the analyses. Applying this criterion, 10 sessions were excluded. A further two sessions (day 4 from R2336, day 8 from R2198) were excluded because of data loss due to the headstages becoming disconnected from the microdrives during the rest session. In total 24 sessions were submitted for further analysis. Only reactivation/replay trajectory events occurring at the two corners were analyzed, as the corners represented locations where the animal had a choice as to which way to turn.

Data analysis

Ratemaps for the Z-track were generated after first excluding areas in which the animals regularly performed non-perambulatory behaviors (e.g., eating, grooming); the final 10cm at either end of the track and 5cm around each of the two corners. Similarly, periods when the animals' running speed was < 10cm/s were also excluded. To generate ratemaps, the animals' paths were linearized, dwell time and spikes binned into 2cm bins and smoothed with a Gaussian kernel (σ = 5bins), firing rates were calculated for each bin by dividing spike number by dwell time. Separate ratemaps were generated for runs in the outbound and inbound directions (mean correlation between inbound and outbound ratemaps: $r = -0.0032$, 1-sample t-test: $p = 0.74$). To identify place fields, spatial bins whose rate exceeded the mean firing rate of the cell on the track were only considered. Hippocampal cells were classified as place cells if they exhibited firing greater than its mean rate for 20 contiguous bins and if the peak firing rate was > 1 Hz. Interneurons, identified by narrow waveforms and high firing rates, were excluded from all analyses. Grid cells were identified using a method adopted previously (Ólafsdóttir et al., 2016). Spikes were manually sorted using Tint (Axona Ltd) spike-sorting software.

Two approaches were employed to analyze place cell reactivations occurring at the two corners (events occurring elsewhere were not analyzed). The 'reactivation' analysis was used to identify which of the arms making up the track was most strongly represented during events, and the replay 'trajectory' analysis identified reactivations of previously taken paths. The methods overlapped significantly for the two approaches, the main difference being the way we carried out the position decoding.

Putative reactivation events were identified based on the activity of hippocampal place cells using a similar method to Pfeiffer and Foster (2013) and Ólafsdóttir et al. (2016). To identify reactivation events, multi-unit (MU) activity from CA1 place cells were binned into 1ms temporal bins and smoothed with a Guassian kernel (σ = 5ms). Periods when the MU activity exceeded the mean rate by 3 standard deviations were identified as candidate reactivation events. The start and end points of each candidate event were determined as the time when the MU activity fell back to the mean. Events less than 40ms long were rejected. Further, events were excluded if the animals' movement speed during the event exceeded 3cm/s or if the animals were located away from the two corners (total number of events = 4425). Event detection for the replay trajectory analysis was identical to that for the arm reactivation analysis except we included an additional cell activity criteria for selecting events. Namely, at least 15% of the place cell ensemble or more than 5 place cells, whichever was higher, needed to be active during an event for it be included for analyses.

For position decoding of reactivations, we summed the number of spikes per cell for each event, and a Bayesian framework (Davidson et al., 2009; Ólafsdóttir et al., 2016; Zhang et al., 1998) was used to calculate the probability of the animal's position in each spatial bin given the observed spikes; the posterior probability matrix. Note, two posterior probability matrices were generated for each event, one for inbound runs and one for outbound runs. We then summed the probabilities for each arm (a total of 6 arms given the outbound and inbound runs were decoded separately), to identify which arm was being expressed most strongly during the events.

A similar framework was applied to calculate the posterior probability matrices used for analysis of full replay trajectories, with the following exceptions. Spike data were divided up into 10ms temporal bins and a probability distribution over position was calculated for each temporal bin. The distribution corresponding to each temporal bin was normalized such that is summed to one. Temporal bins containing no spikes were set to zero and hence did not contribute to either the trajectory fitting, in the case of place cells, or coherence measure, in the case of grid cells.

To score the extent to which putative trajectory events represented a constant speed trajectory along the linearized Z-track we applied a line-fitting algorithm (Ólafsdóttir et al., 2016). Lines were defined with a gradient (V) and intercept (c), equivalent to the velocity and starting location of the trajectory. The goodness of fit of a given line (R(V,c)) was defined as the proportion of the probability distribution that lay within 30cm of it. Specifically, where P is the probability matrix:

$$R(V,c) = \frac{1}{n}\sum_{t=0}^{n-1} P(|x(t) - (V.t.T + c)| \leq d) \quad (1)$$

where t indexes the time bins of width T and d is set to 30cm. R(V,c) was maximized using an exhaustive search to test all combinations of V between -50 m/s and 50 m/s in 0.5 m/s increments (excluding slow trajectories with speeds > -2 m/s and < 2 m/s) and c between -15 m and 21 m in 0.01 m increments. This goodness of fit value was then normalized by dividing it by the summed probability in the matrix (n.b. bins with no spikes were set to 0).

To assess candidate replay events for significance we carried out a spatial shuffle of the place cell ratemaps. Specifically, each ratemap was 'rotated' by shifting it relative to the track by a random number of bins drawn from a flat distribution between 1 and the length of the track minus 1 bin. The ratemap for each cell was rotated independently and in each case trailing bins were wrapped around to ensure an equal number of bins were used for each shuffle. This process was repeated 100 times for each event and for each shuffle we recalculated a goodness of fit measure (as described above). This enabled us to estimate the probability of obtaining a given event by chance. Replay trajectory events were defined as those with an individual p value below 0.025 (a total of 513 trajectory events). Shuffle and data distributions were compared using a 2-sample Kolmogorov-Smirnov test.

A similar approach was used to decode the animals' locations during track running, except spikes were binned into 500ms temporal bins and location was decoded from the posterior probability matrix using a simple maximum likelihood method. Within each temporal bin an animal's location was decoded to the bin with the highest posterior probability and this was compared with the known true location (median decoding error for all sessions = 17.5cm, $\sim$3% of track length).

Reactivation analysis

Corner stops were divided into two temporal sections. The first 5 s following an animal's arrival at the corner and the last 5 s preceding an animal's departure from a corner were categorised as 'engaged' periods. Any time in between the two was categorised as 'disengaged' periods. Reactivation events were analyzed separately for the two temporal sections. Temporal sections which lasted < 2 s were excluded. If corner stops lasted less than 10 s, we treated the entire stop as an engaged period.

For track reactivation events we classified events as 'congruent' if the decoded arm belonged to the same run the animal was carrying out at the time of the event. For example, if an animal was carrying out an inbound run and an arm on the inbound run was reactivated. Alternatively, events were classified as 'incongruent'. Furthermore, reactivation events were considered 'local' if they decoded one of the two arms adjacent to the animal. For example, if the animal was located at corner1 during an outbound run, and if arm1 or arm2 were reactivated. Note, for an event to be considered local it also had to be congruent. To estimate the proportion of congruent/local events one would expect to obtain by chance we carried out a cell ID shuffle of each event. Namely, cells active in an event were randomly reassigned to a different cell's ratemap not active in the event and track decoding carried out on this shuffled data. This process was repeated 100 times and the mean proportion of shuffled congruent/local events used as chance level.

For the replay trajectory analysis, we used the same method to categorise events as congruent or incongruent. However, to categorise local events we estimated the mean position during an event and if it lay within 60cm of the animal's actual position (ahead or behind it) we considered the event to be local. We used an alternative approach to analyze the physical proximity of replay trajectory events. Namely, we calculated the distance between the animal and the start as well as the end of the decoded replay trajectory. Furthermore, we categorised events as 'forward' and 'reverse' on the basis of the gradient of the line fit to the place cell posterior probability matrix. Forward events being characterized by a positive gradient which represent reactivation of place cells in the same sequence as they were experienced on the track. Reverse events, characterized by a negative gradient, corresponding to reactivation of place cells in the opposite order to which they would normally be active during running. Finally, events representing positions ahead of the animal were categorised as prospective. To estimate the proportion of congruent/local trajectory events one

would expect by chance we randomly reassigned events to either engaged or disengaged periods 100 times, and computed the mean proportion of local and congruent events from the shuffled data. For forward versus reverse classification we used 50% as chance level.

Finally, events preceding correct turns at the corners were categorised as correct events and those preceding incorrect turns error events.

To analyze the temporal dynamics of congruent/local reactivations during corner stops we divided each corner period into 2 s time bins, and computed the proportion of congruent/local events for each bin up to 30 s following corner arrival/prior to corner departure. For this analysis, we only included corner stops that were at least 30 s long. To estimate whether the obtained proportion for each time bin was significantly above chance we binned the data into 5 s time bins, advancing in 2.5 s increments, and for each bin calculated whether the obtained proportion was significantly greater than chance level.

We carried out a number of control analyses to ascertain the differences obtained for different levels of task engagement could not be accounted for by different LFP states underlying periods associated with task engagement and disengagement. First, to equate the movement speeds during engaged and disengaged events we applied the following approach. Data from the disengaged periods was subsampled to have the same median speed as the engaged periods – this was achieved by removing data corresponding to the highest or lowest speed points first. Second, we excluded replay trajectory events whose total length was less than 2 m (thereby removing replay trajectories that might results from theta phase precession or sequences), following O'Neill and colleagues (O'Neill et al., 2017). Third, we limited the reactivation events to those whose log(theta/delta) ratio was at least one standard deviation below the mean log(theta/delta) ratio measured during movement (> 10cm/s). Fourth, we limited replay trajectory events to those which overlapped with a detected ripple (150-250Hz) event (see details of ripple detection in the section below).

Grid coherence analysis

To analyze coherence between spatial representations of place and grid cells during full hippocampal replay trajectories we followed a method we had adopted previously (Ólafsdóttir et al., 2016). Briefly, we applied the same Bayesian framework to the grid cell spikes as we did for the place cell spikes. Hence, for each replay event we also calculated a posterior probability matrix based solely on the observed grid cell spikes. Rather than fitting straight-line trajectories to the periodic grid cell posteriors, we compared the best-fit line from the concurrently recorded place cell posterior. Specifically, we fitted a line with the same intercept and slope as the concurrent place cell event and calculated the proportion of the probability distribution lying within x/2cm of the line and divided this by the total probability in the matrix (again, empty bins were set to 0). Where x was equal to the average size of the grid cell firing fields recorded from that animal on the linear track. This value we used to index grid-place cell replay coherence. To estimate statistical significance of the observed coherence scores we used the following shuffling procedure: each grid cell posterior was randomly paired with 100 non-concurrent place cell events from the same animal and from the same session. The line fitting procedure to estimate grid-place cell replay coherence, described above, was then re-run on the randomly paired events. If the grid-place cell coherence score obtained in the original data exceeded the 97.5th percentile of its shuffle distribution we deemed the coherence to be better than chance.

To test whether grid-place cell coherence differed between engaged and disengaged periods we carried out the bootstrapping procedure, described in the 'Statistics' section below.

Local field potential analysis

Local field potential (LFP) from CA1 was recorded at 4.8kHz throughout the experiment. To analyze sharp-wave ripples (Buzsáki et al., 1992) and theta-band (O'Keefe and Nadel, 1978; Vanderwolf, 1969) oscillation the LFP was first down-sampled to 1.2kHz and then band-pass filtered between 150 and 250Hz and 6-12Hz, respectively. An analytic signal was constructed using the Hilbert transform, taking the form:

$$s_a(t_k) = s(t_k) + i\mathrm{H}[s(t_k)] \tag{2}$$

where H specifies the Hilbert transform, s(tk) is the filtered LFP signal, tk = kΔ, where k = 1,...,K indexes the time-step and Δ is the inverse of the sampling rate. An instantaneous measure of power was found by taking the squared complex modulus of the signal at each time point.

For the track-reactivation analysis we down-sampled this measure to 50Hz to match the position sampling rate. Furthermore, to obtain a more stable measure for theta power we computed the ratio between theta and delta (2-4Hz) power for each recording sample and used the log-transformed theta/delta ratio as an estimate of instantaneous theta power (Jackson et al., 2006). For each reactivation event, we computed the mean power in the log(ripple/theta) ratio band. For ripple event detection, we identified periods where the ripple power exceeded 2.5std above the mean. The start and the end of a ripple event was marked by the point when the power crossed the mean. Events lasting less than 40ms or more than 500ms were excluded and events separated by less than 40ms were joined together. To assess LFP coherence between CA1 and deeper layers of the MEC we used the mscohere function in MATLAB 2014a (Mathworks. MA). Coherence was analyzed for frequencies up to 200Hz. We also carried out an alternative LFP analysis using a method described by Masimore et al. (2004). Briefly, to analyze hippocampal LFP self-coherence we obtained the LFP

spectrogram using the spectrogram MATLAB function (Mathworks, MA) and then correlated each frequency with itself and all other frequencies. A similar method was used for analyzing coherence between hippocampal and MEC LFP, except in this instance correlations were done between hippocampal and MEC LFPs.

R2142 was excluded from the LFP analyses due to problems with EEG recordings.

Model based prediction

Binary classification decision trees, a simple form of classifier, were used in order to predict, based on the preceding reactivation, if an animal's next turn would be correct or incorrect. First, because of the disparity in the number of error and correct events, correct events were randomly subsampled to match the number of error events; the procedure was repeated 10 times, model training and testing being completed independently for each iteration. Next, the data used to fit and test each model was segregated further using 10-fold cross validation: reactivation events were randomly divided into 10 equally sized groups, on any given 'fold' 9 of these groups were used to train the model and the remaining group used to generate predictions, this process was iterated 10 times such that each event was used once to make and test a prediction. The model was fit using the fitctree function in MATLAB 2016b (Mathworks, MA). Three predictor variables based on the content of the replay were used: (1) how congruent and, (2) how local each event was, as well as (3) the average ripple band power (150-250Hz) of the LFP during the event. For use in the model only, the congruence and locality of events were described using continuous variables calculated as follows. Congruence measure: For each event the posterior probability over position was calculated in the same way as it was for the reactivation analysis. The probabilities for the congruent and incongruent runs were each summed to give two numbers, P_c and P_i respectively. The congruence measure was then defined as:

$$\text{Congruence} = \frac{P_c - P_i}{P_c + P_i} \tag{3}$$

Hence, a congruent event would yield a positive score. Locality measure: Again, the posterior probability over position corresponding to the congruent and incongruent runs were used. The portions of both probability distributions corresponding to the two arms of the maze adjacent (i.e., local) to the animal's current position were identified and summed to give a single number, P_l. The same procedure was applied for the arm that was remote to the animal's location yielding a value P_r. The locality measure was then defined as:

$$\text{Locality} = \frac{P_l - P_r}{P_l + P_r} \tag{4}$$

The locality measure was calculated for all events regardless of their congruence.

Predictions were generated by applying the parameters of the trained decision tree to the remaining test data using the MATLAB function predict. Prediction accuracy was defined simply as the total proportion of events that were classified correctly. To establish significance, the same training and testing procedure was applied to shuffled datasets. Each shuffled dataset was generated by randomly reallocating the response variable (i.e., correct/error) relative to the predictor variables (i.e., congruence, locality, ripple power); the relationship between the predictors was not permuted (e.g., congruence values were not shuffled relative to locality and ripple power). This process was repeated 100 times for each of the 10 subsampled iterations, resulting in a distribution of 1,000 prediction accuracies, against which the true prediction accuracy was ranked to generate a p value.

Histology

Rats were anaesthetised (4% isoflurane and 4L/min O2), injected intra-peritoneal with an overdose of Euthatal (sodium pentobarbital) after which they were transcardially perfused with saline followed by a 4% paraformaldehyde solution (PFA). Brains were carefully removed and stored in PFA which was exchanged for a 4% PFA solution in PBS (phosphate buffered saline) with 20% sucrose 2-3 days prior to sectioning. Subsequently, 40-50 μm frozen sections (coronal for CA1 and sagittal for MEC) were cut using a cryostat, mounted on gelatine-coated glass slides, stained with cresyl violet and cleared with a clearing agent (Histo-Clear II). Images of the sections were acquired using an Olympus microscope, Xli digital camera (XL Imaging Ltd.). Sections in which clear tracks from tetrode bundles could be seen were used to confirm CA1 and MEC recording locations.

QUANTIFICATION AND STATISTICAL ANALYSIS

To assess differences in the proportion of different event types (e.g., congruent events) for engaged and disengaged periods we bootstrapped the data and computed the 95% confidence interval. Namely, we resampled the data with replacement 10,000 times, each time calculating the proportion of a given event type for a particular event period. We then subtracted the proportion of events of a given type occurring during disengaged periods from that occurring during engaged periods, and if 97.5% of the difference scores exceeded 0 we deemed the result significant. To estimate if the obtained proportion significantly differed from chance we counted the number of times the bootstrapped data exceeded the empirically derived chance level (for details of chance calculation see 'Reactivation analysis' section above), if more than 97.5% of the bootstrapped data was greater than chance we deemed the data to be significantly above chance. When comparing data and shuffle distributions we used a 2-sample Kolmogorov-Smirnov test. When

comparing LFP power and grid-place cell replay coherence during engaged and disengaged periods we carried out the same analysis, but for each bootstrap iteration we computed means rather than proportion. All correlations were carried out using the Pearson product-moment correlation coefficient.

To assess whether there was a significant interaction between task engagement and decision accuracy at the corners we carried out the following analysis. We bootstrapped the data for engaged, disengaged, correct and incorrect events separately, obtaining a bootstrapped distribution of %congruent/%local reactivations for each of the four categories (as described above). For each correct and incorrect bootstrapped distribution pair, we computed a difference distribution (by subtracting the correct distribution from the incorrect distribution). We then compared the engaged and disengaged difference distributions to assess whether the engaged difference scores were significantly higher than the disengaged difference scores; implying future decision accuracy modulates the content of place cell reactivations more for reactivations occurring during engaged periods, compared to disengaged periods. If more than 97.5% of the engaged difference scores exceeded the disengaged difference scores we deemed the interaction significant.

All statistics, including definition of center, dispersion, precision measures and exact values of n, are reported in the Results section of the manuscript as well as in the figure legends.

DATA AND SOFTWARE AVAILABILITY

The data that support the findings of this study are available from the corresponding authors upon request.

Neuron, Volume *96*

Supplemental Information

Task Demands Predict a Dynamic Switch in the Content of Awake Hippocampal Replay

H. Freyja Ólafsdóttir, Francis Carpenter, and Caswell Barry

Animal	Experimental Day	#Place cells	Outbound error (cm)	Inbound error (cm)	Mean Error (cm)	% Replay	p-value (KS test, data vs shuffle)
R2142	4	37	22	10	17	37.54	1.05×10^{-29}
	5	46	20	12	16	24.16	6.52×10^{-19}
	6	39	30	24	27	46.34	7.29×10^{-5}
R2192	4	34	20	16	18	40.46	1.21×10^{-34}
	5	39	16	12	14	39.64	8.12×10^{-83}
	6	70	12	12	12	43.09	1.77×10^{-55}
	7	68	10	10	10	56.25	6.77×10^{-45}
	8	72	10	10	10	59.35	2.32×10^{-32}
R2198	9	30	20	12	16	32.26	1.72×10^{-50}
	10	27	12	12	12	29.79	4.61×10^{-8}
R2217	5	25	16	16	16	23.13	1.81×10^{-12}
	7	40	14	18	16	31.00	5.25×10^{-12}
R2242	1	30	18	22	18	27.43	4.03×10^{-36}
	4	21	24	18	22	70.00	0.00072
R2335	3	34	30	26	28	11.11	4.44×10^{-6}
R2336	2	25	24	20	22	19.86	3.45×10^{-9}
	3	39	24	26	26	45.90	3.76×10^{-10}
	5	34	16	28	20	7.70	0.0013
	6	67	12	14	14	39.67	2.28×10^{-13}
R2337	2	50	22	14	18	27.82	3.97×10^{-37}
	3	50	20	16	18	40.35	5.88×10^{-32}
	4	71	18	16	16	40.84	1.98×10^{-75}
	5	48	28	22	24	44.81	3.58×10^{-59}
	6	46	26	20	22	23.53	3.58×10^{-20}
Median		34	20	16	17.5	38.59	

Table S1. Related to Figure 1. Place Cell Number, Decoding Errror and Replay for Each Experimental Day

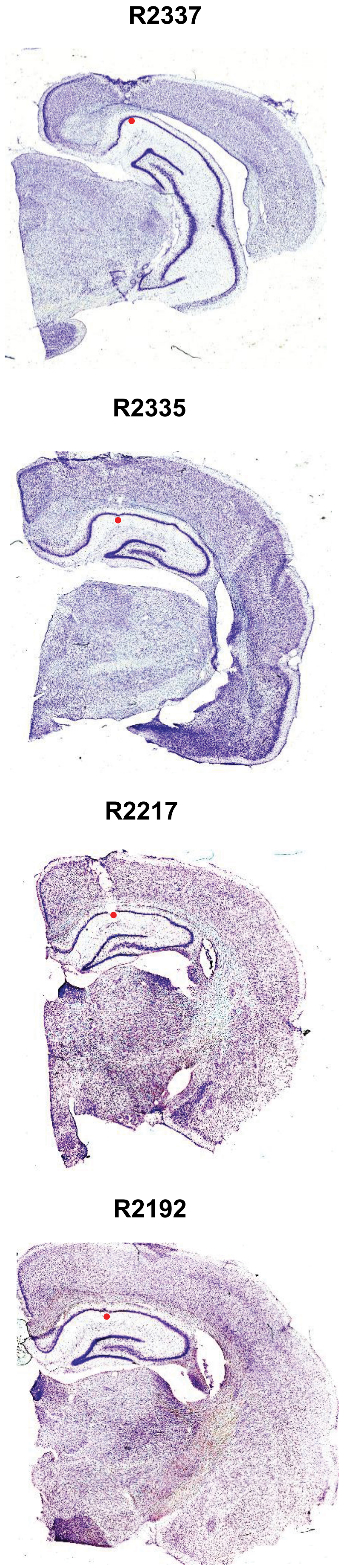

Figure S1. Related to Figure 1. Tetrode Locations. Four representative examples of Cresyl violet stained sections showing the tetrode tracks from coronal sections. Red circle indicates the recording location for data included in this study. Title shows rat ID.

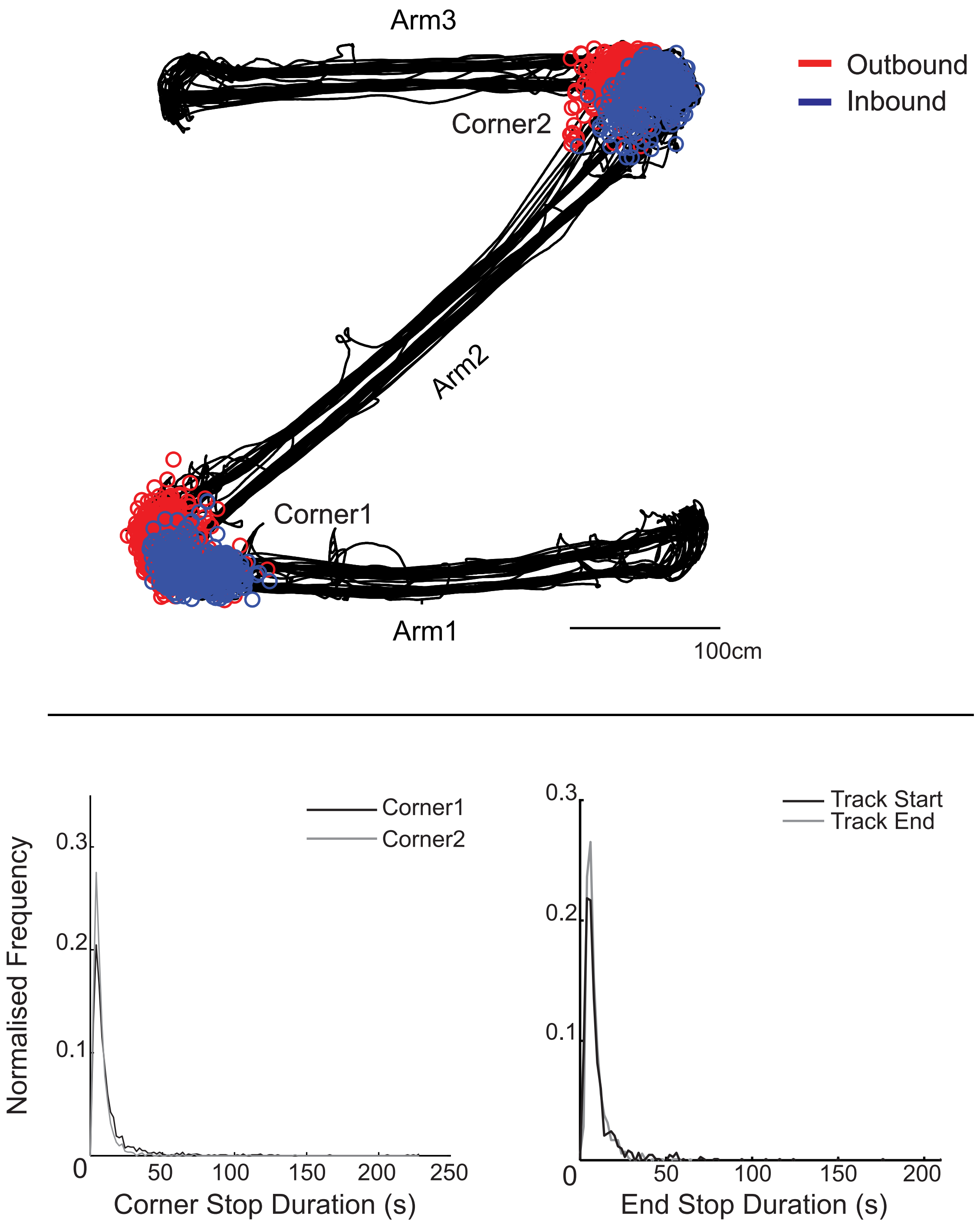

Figure S2. Related to Figure 1. Position and Duration of Corner Stops. Top: The location of animals during corner periods for outbound (Arm1 -> Arm3) and inbound (Arm3 -> Arm1) runs are indicated by the coloured circles. Each circle indicates mean position during a single corner period. Black line - path from lone recording session (i.e. from a single animal), included to illustrate the track extent. Note, primary tracking LED was offset slightly (i.e. 7cm) from the side of animals' heads - hence, outbound and inbound runs appear to not overlap. Bottom: Left: distribution of stopping period durations at the two corners. Right: distribution of stopping period durations at the two track ends (track start = beginning of arm1; track end = end of arm3).

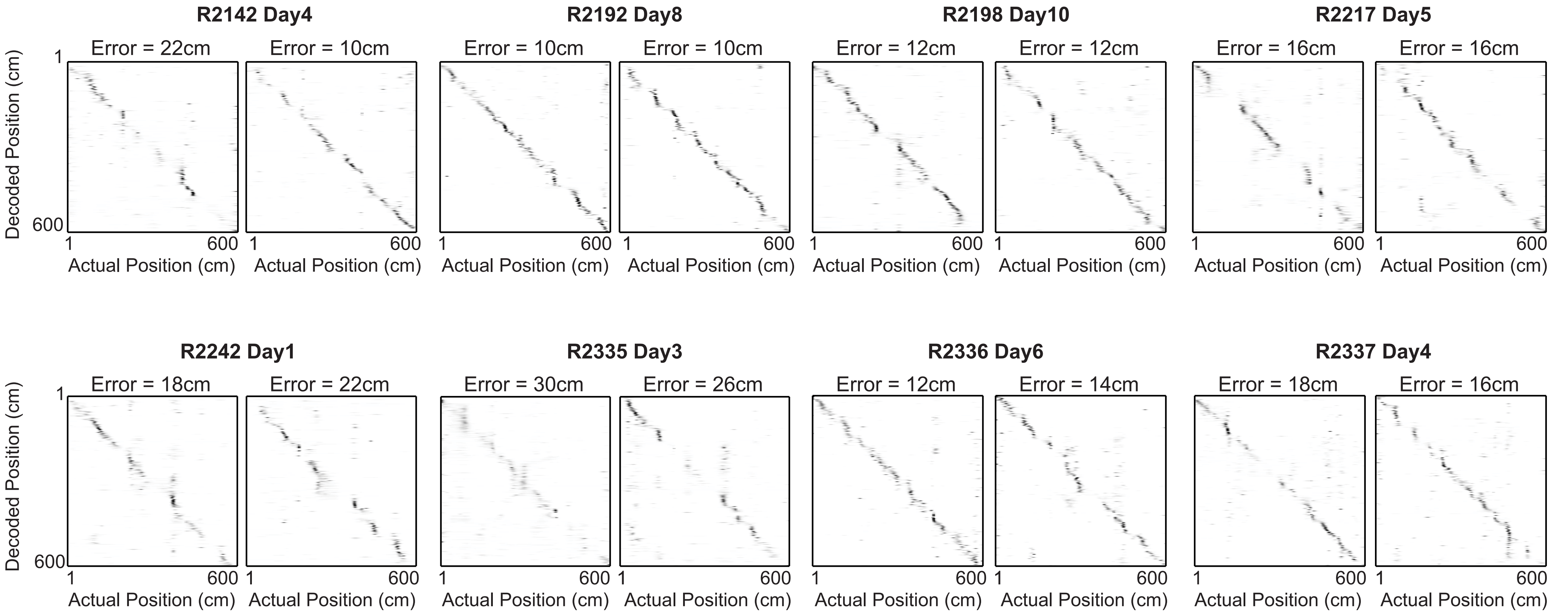

Figure S3. Related to Figure 1. Accuracy of Online Decoding. Confusion matrices for position decoding carried out on spike data while the animals ran (>10cm/s) on the Z-track. A representative session was selected for each animal. Right: confusion matrices for outbound runs, left: confusion matrices for inbound runs. Darker shades indicate higher probability of position. x-axis: actual position of rat (cm), y-axis: decoded position (cm).

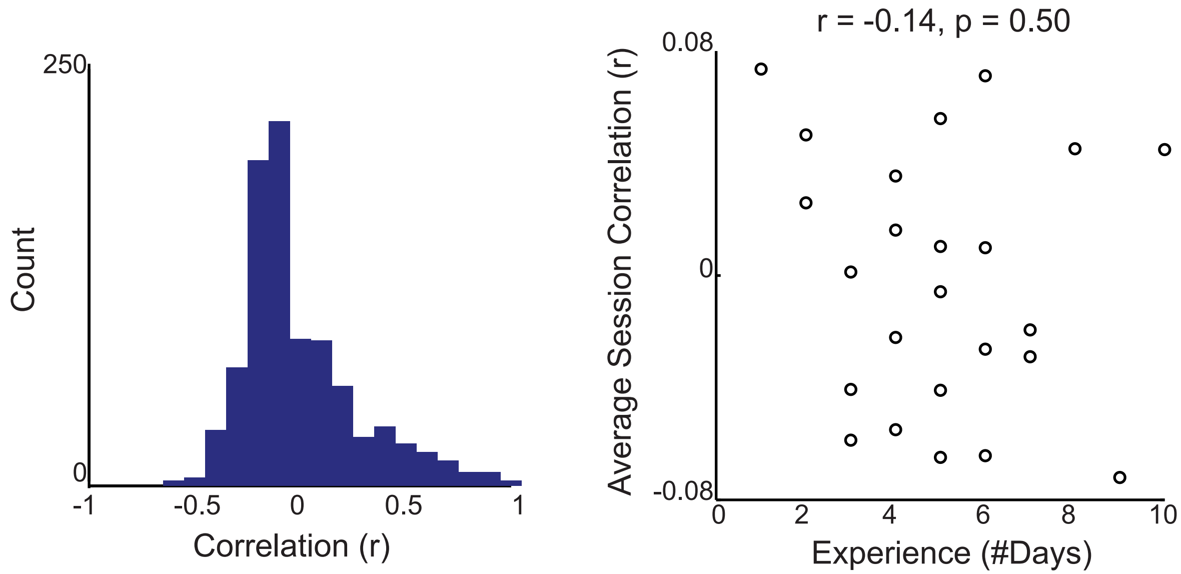

Figure S4. Related to Figure1. Place Cell Activity Is Directionally Modulated on the Z-track. Left: distribution of spatial correlations (r) between inbound and outbound ratemaps for all cells recorded on the track. Right: Mean spatial correlation for each session vs. animals' experience of the track.

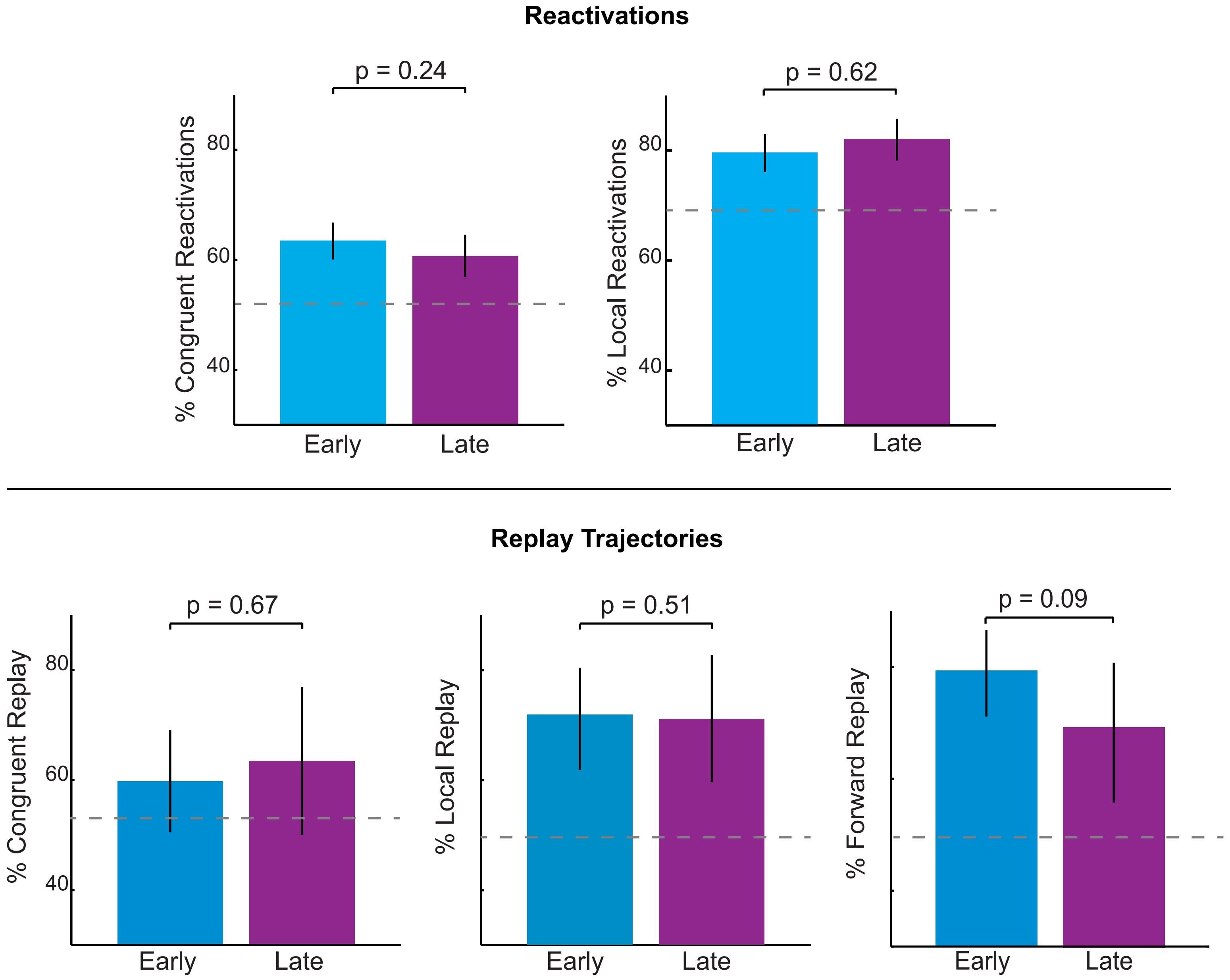

Figure S5. Related to Figure 2. Replay for Early and Late Engaged Periods of Corner Stops. Upper panel: Proportion of reactivation events classified as congruent (left) or local (right) for early (blue) and late (purple) temporal sections of engaged periods at corner stops. Lower panel: proportion of replay events classified as congruent (left), local (middle) or forward (right) for early and late temporal sections. Error bars indicate 95% confidence interval based on bootstrapped data. Dashed horizontal line shows chance.

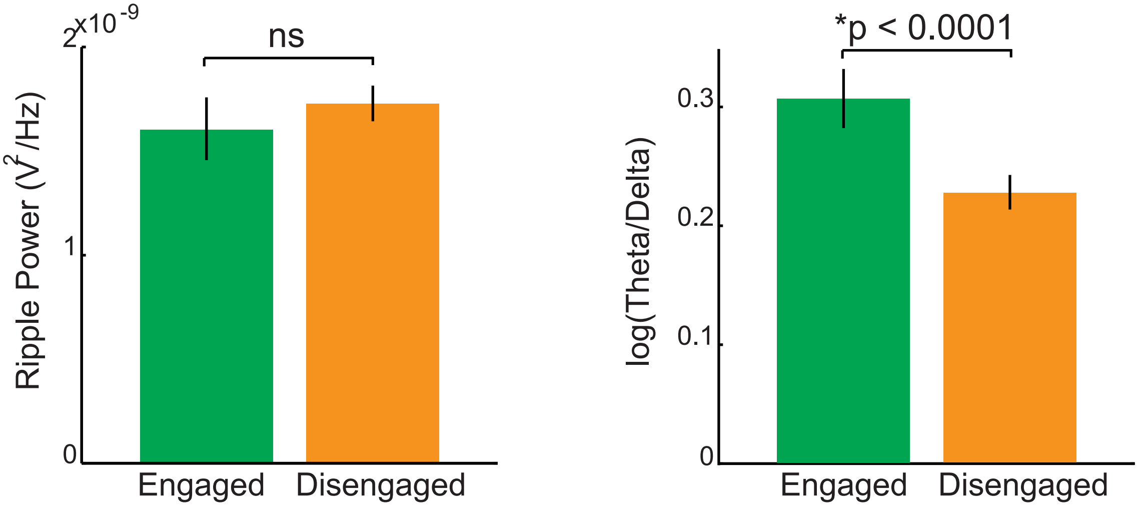

Figure S6. Related to Figure 2. Power in the Theta and Ripple Band During Reactivations. Left: mean power in the ripple band (150-250Hz) for engaged (green) and disengaged (amber) events. Right: same as left but for log(theta/delta) ratio, where theta band is 6-12Hz and delta band is 2-4Hz. Error bars show 95% confidence interval based on bootstrapped data.

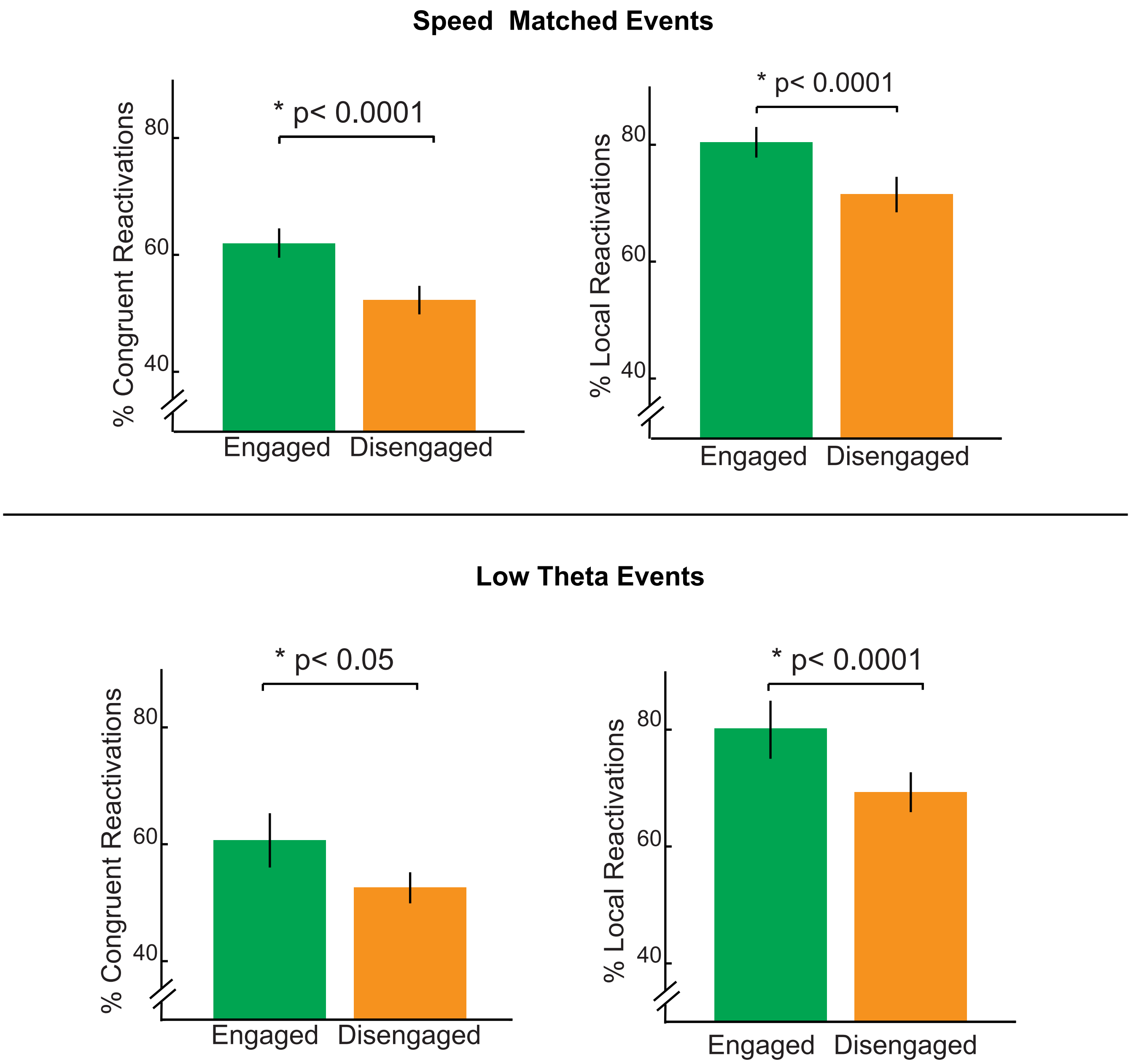

Figure S7. Related to Figure 2. Effect of Task Engagement Over Reactivations is Not an Artefact of Speed or High Theta During Events. Upper panel(left): Proportion of events classified as congruent for engaged (green) and disengaged (amber) periods after excluding events in order to match the median speeds of the two categories. Right: same as left but for local reactivations. Before excluding events median engaged speed = 1.80cm/s, median disengaged speed = 1.13cm/s. Bottom panel: same as upper but after the exclusion of events with high theta power (i.e. theta power < mean theta power during movement -1SD, mean = 0.64, SD = 0.42, threshold = 0.22). Mean engaged log(theta/delta) power = 0.31, mean disengaged log(theta/delta) power = 0.23. Error bars show 95% confidence interval based on bootstrapped data.

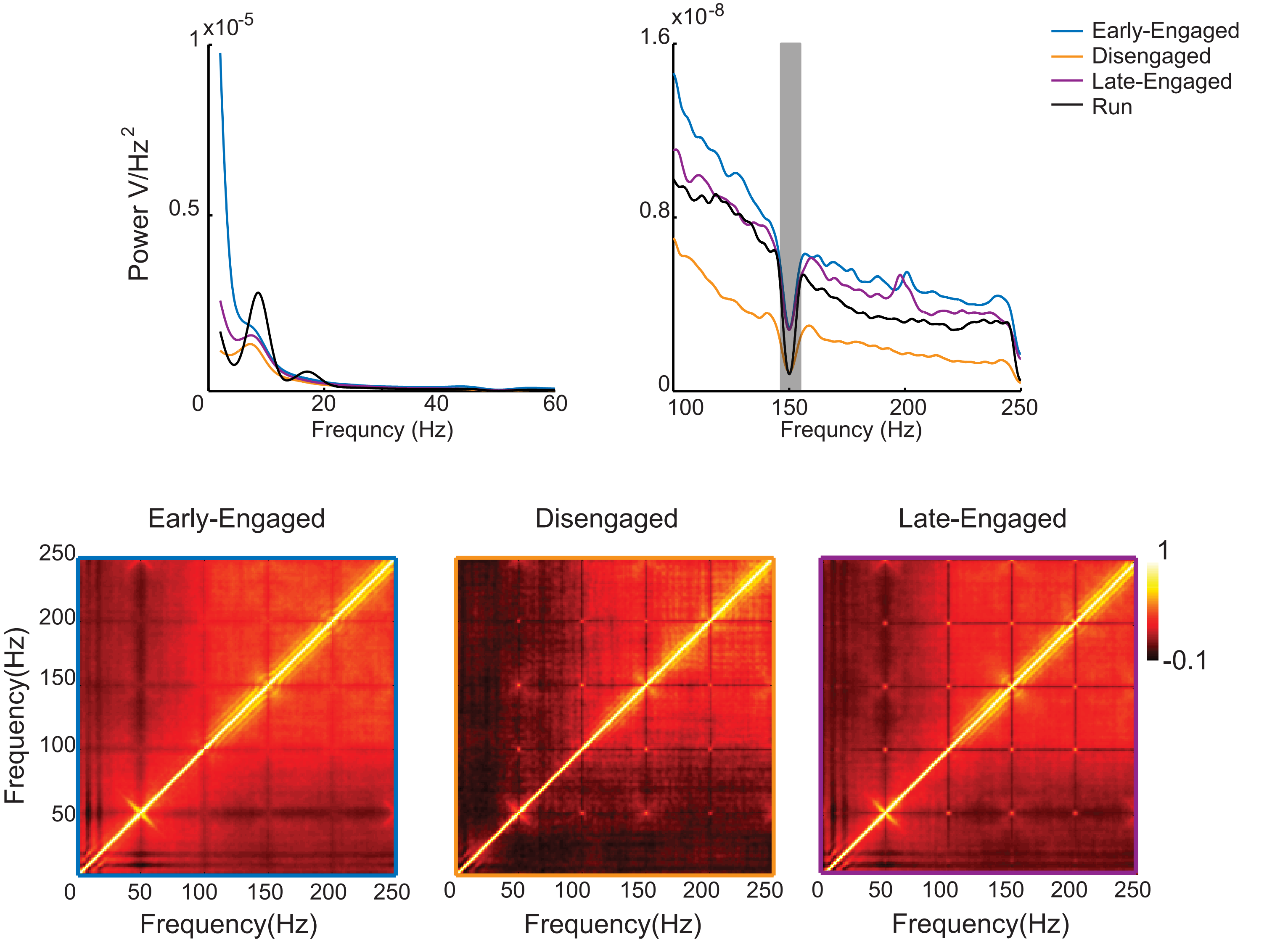

Figure S8. Related to Figure 2. Power-Spectral Density (PSD) and Self-Coherence Analysis for Distinct Temporal Sections of Corner Stops. Top left: PSD for early-engaged (blue), disengaged (amber), and late-engaged (purple) sections of corner stops as well as during periods of movement (black) for low (1-60Hz,left) and high (100-250Hz, right) frequencies. Bottom: self-coherence (Masimore et al. 2004) plots for the three temporal sections at the corners. Lighter colours indicate higher correlations. A notch filter was applied at multiples of 50Hz to eliminate electrical mains noise.

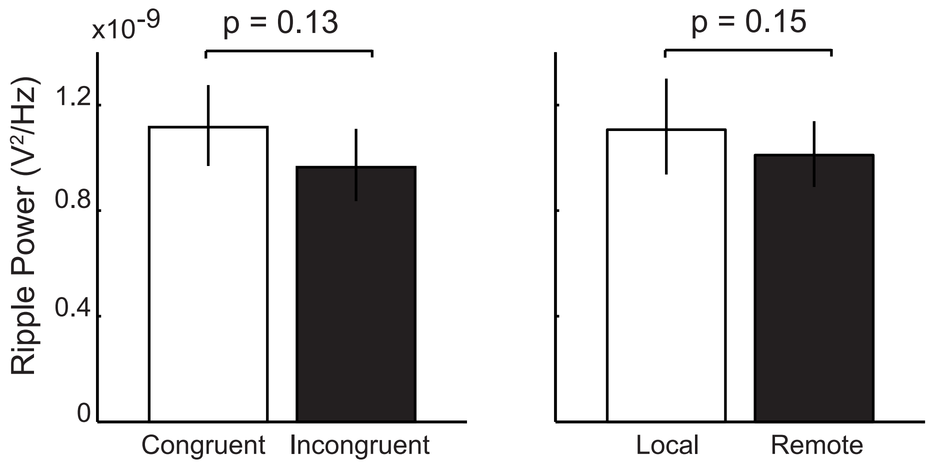

Figure S9. Related to Figure2. Ripple Power Does Not Differ Between Reactivation Categories. Power in the ripple band (150-250Hz) for congruent and incongruent reactivation events (left) and local and remote (right) events. Error bars indicate 95% confidence interval based on bootstrapped data.

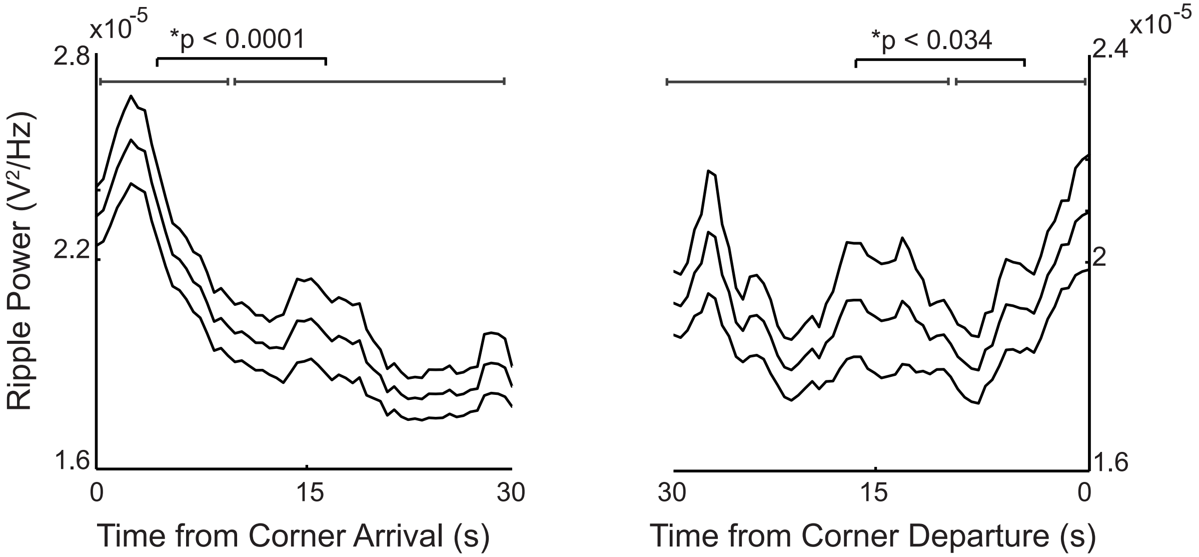

Figure S10. Related to Figure 2. Ripple Power During Corner Stops. Power in the LFP ripple band (150-250Hz) as a function of time from arrival (left) and departure (right) from a corner. x-axis: time (s), y-axis: ripple power (V^2/Hz). Power in the first 10 seconds after arrival and before departure was higher than power in the 20-30 second period. Error bars show 1SD of the data.

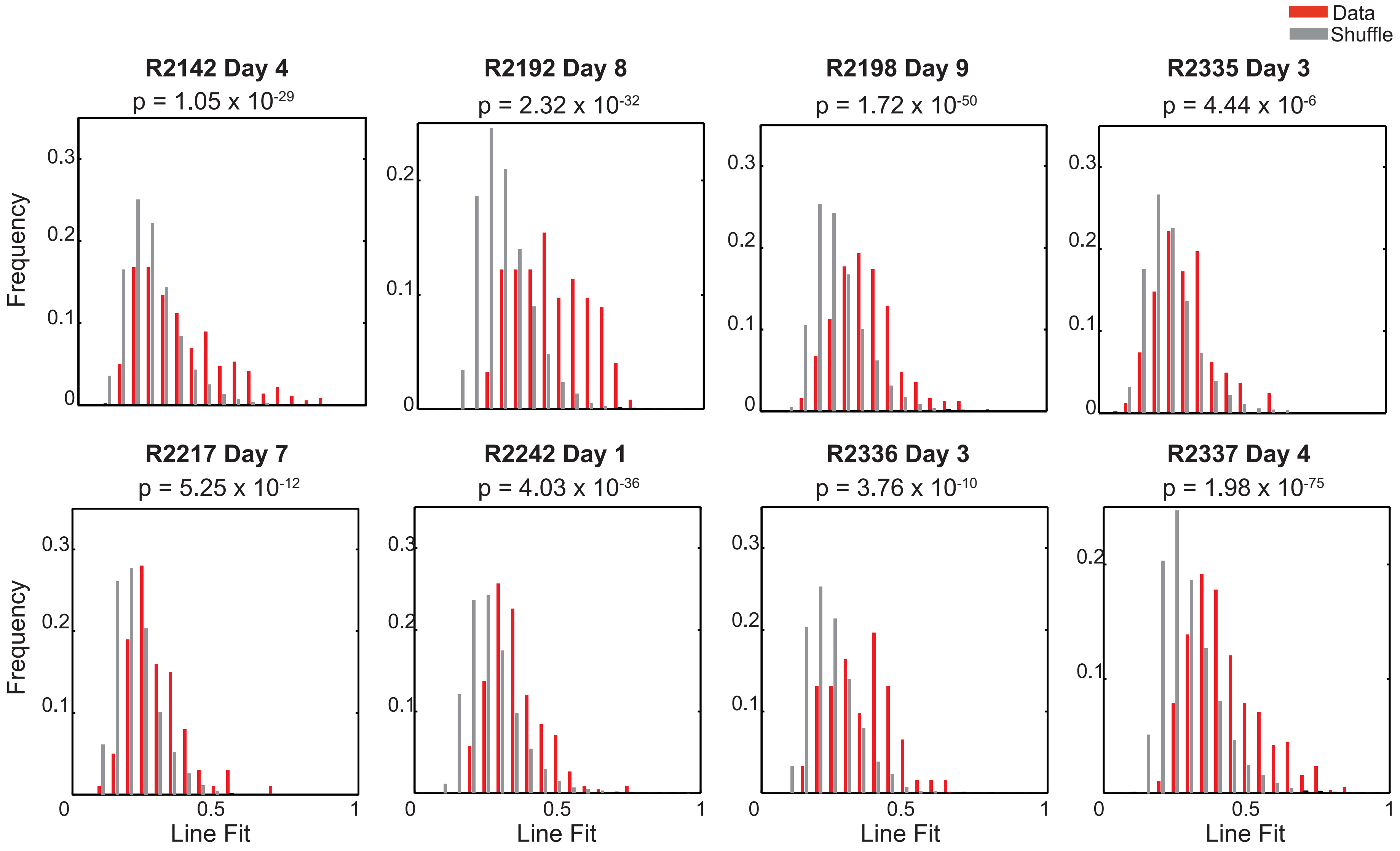

Figure S11. Related to Figure 3. Histogram of Best-Fit Lines for Replay Events. Representative examples, from each animal, of frequency distributions of best-fit lines for replay events recorded in a given session (red) against that for shuffled data (grey). Title shows p-value of a Kolmogorov-Smirnov test comparing the data and shuffle distributions. The data and shuffle distributions were normalised by dividing by the total number of events in each.

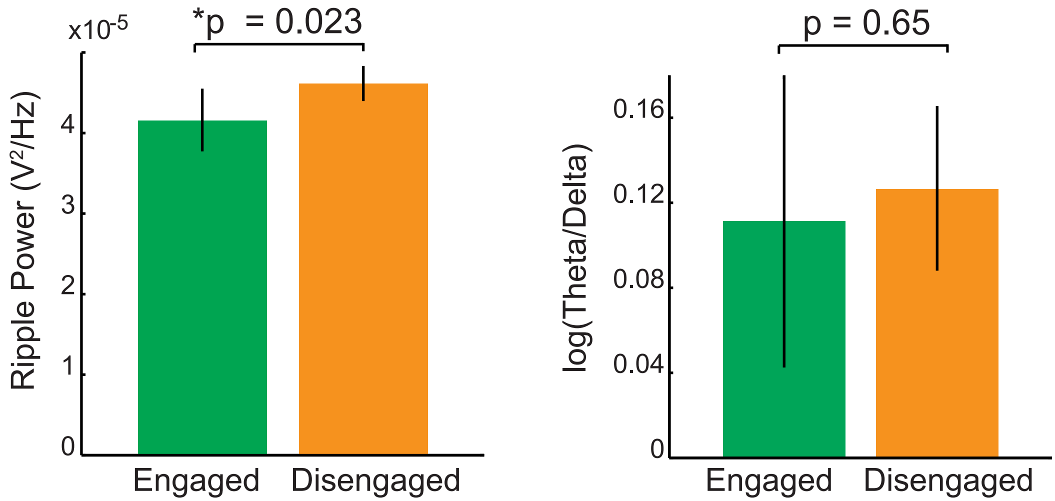

Figure S12. Related to Figure 3. Ripple and Theta Power During Replay Events. Left: Mean power in the ripple band (150-250Hz) during engaged (green) and disengaged (amber) replay events. Right: same as left but for log(theta/delta) ratio, where theta band is 6-12Hz, and delta band 2-4Hz. Error bars indicate 95% confidence interval based on bootstrapped data.

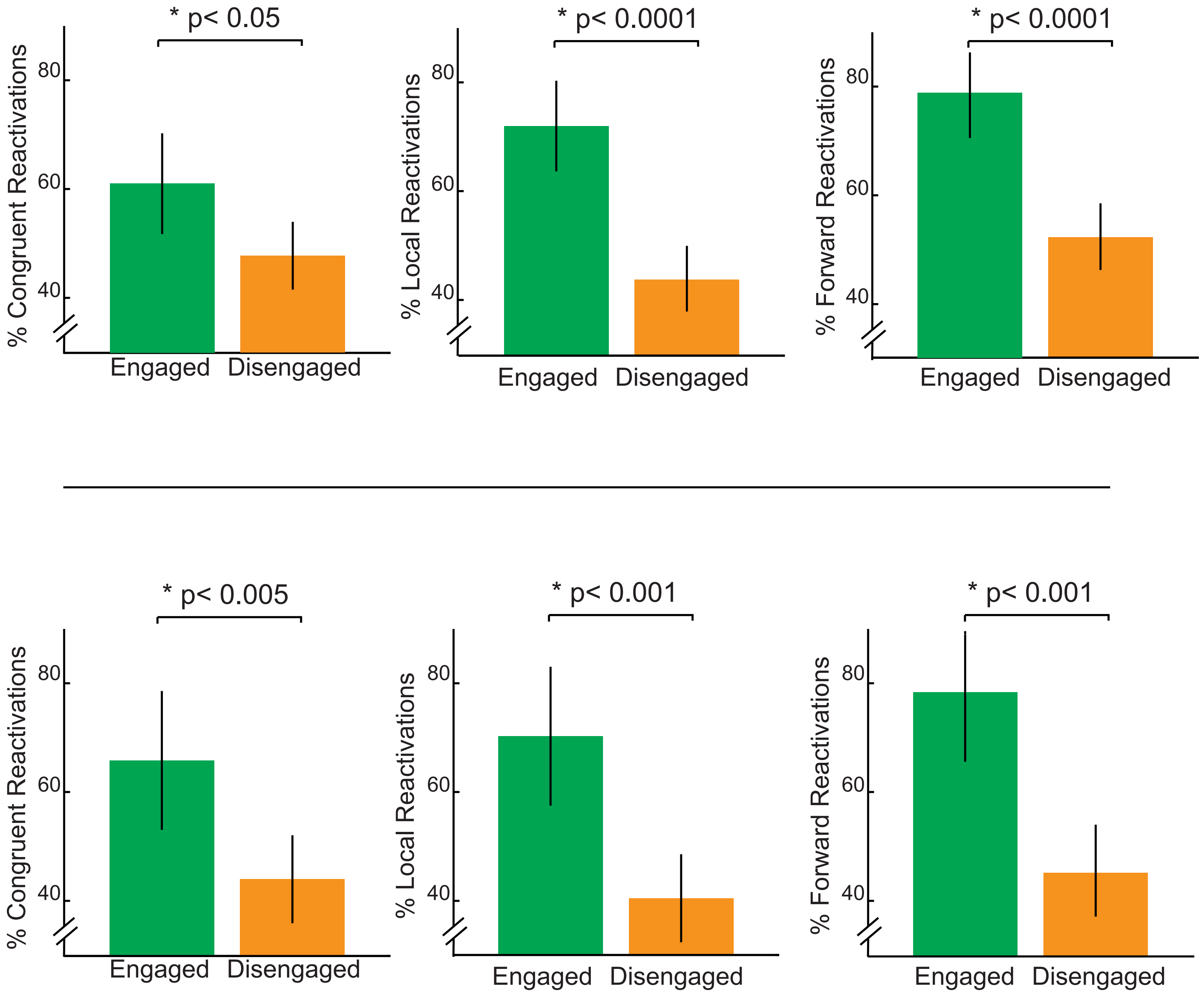

Figure S13. Related to Figure 3. Effect of Task Engagement on Replay After Limiting Analysis to Long Trajectory Events and Events Co-Occurring with Ripples. Upper panel**:** Proportion of events classified as congruent (left), local (middle) or forward (right) for engaged (green) and disengaged (amber) periods after excluding events with a shallow (replay trajectories >2m long) slope (mean engaged slope = 8.7m/s, mean disengaged slope = 7.7m/s). Lower panel: same as upper panel but once analysis was limited to events co-occurring with ripples (38.84% of engaged events, 40.12% of disengaged events). Error bars indicate 95% confidence interval based on bootstrapped data.

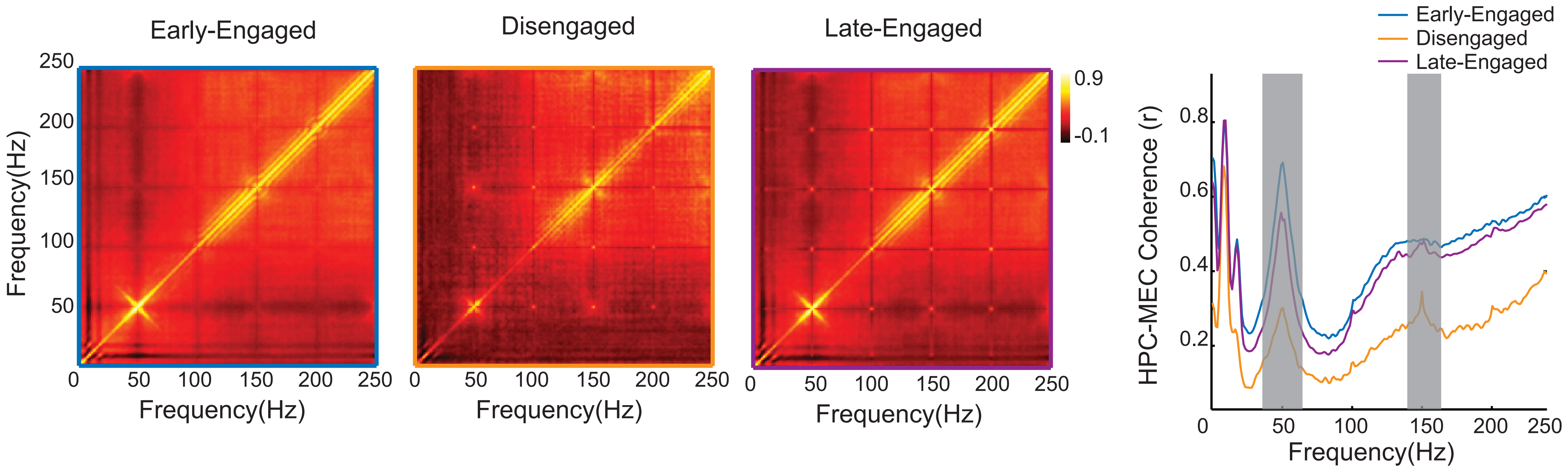

Figure S14. Related to Figure 4. Hippocampal-MEC LFP Coherence During Engaged and Disengaged Replay. Left: Cross-coherence (Masimore et al. 2004) between hippocampal and MEC LFP during early-engaged, disengaged and late-engaged sections of corner stops. Lighter colours indicate higher correlations. Right: Hippocampal-MEC LFP coherence during the engaged-early (blue), disengaged (amber) and engaged-late (purple) sections. Grey area covers a portion of the LFP band contaminated by mains (50Hz) noise.
